# Hormonal Signal Amplification Mediates Environmental Conditions during Development and Controls an Irreversible Commitment to Adulthood

**DOI:** 10.1371/journal.pbio.1001306

**Published:** 2012-04-10

**Authors:** Oren N. Schaedel, Birgit Gerisch, Adam Antebi, Paul W. Sternberg

**Affiliations:** 1 Howard Hughes Medical Institute and Division of Biology, California Institute of Technology, Pasadena, California, United States of America; 2 Max-Planck-Institute for Biology of Ageing, Koeln, Germany; 3 Baylor College of Medicine, Huffington Center on Aging, Houston, Texas, United States of America; Massachusetts General Hospital Havard Medical School, United States of America

## Abstract

Many animals can choose between different developmental fates to maximize fitness. Despite the complexity of environmental cues and life history, different developmental fates are executed in a robust fashion. The nematode *Caenorhabditis elegans* serves as a powerful model to examine this phenomenon because it can adopt one of two developmental fates (adulthood or diapause) depending on environmental conditions. The steroid hormone dafachronic acid (DA) directs development to adulthood by regulating the transcriptional activity of the nuclear hormone receptor DAF-12. The known role of DA suggests that it may be the molecular mediator of environmental condition effects on the developmental fate decision, although the mechanism is yet unknown. We used a combination of physiological and molecular biology techniques to demonstrate that commitment to reproductive adult development occurs when DA levels, produced in the neuroendocrine XXX cells, exceed a threshold. Furthermore, imaging and cell ablation experiments demonstrate that the XXX cells act as a source of DA, which, upon commitment to adult development, is amplified and propagated in the epidermis in a DAF-12 dependent manner. This positive feedback loop increases DA levels and drives adult programs in the gonad and epidermis, thus conferring the irreversibility of the decision. We show that the positive feedback loop canalizes development by ensuring that sufficient amounts of DA are dispersed throughout the body and serves as a robust fate-locking mechanism to enforce an organism-wide binary decision, despite noisy and complex environmental cues. These mechanisms are not only relevant to *C. elegans* but may be extended to other hormonal-based decision-making mechanisms in insects and mammals.

## Abstract 2

A dual mechanism regulates the developmental fate choice in *C. elegans* in response to population density: variation of the threshold of DA hormone required to commit to a certain fate and a positive feedback loop that amplifies this hormonal signal to ensure an organism-wide developmental fate choice.

## Author Summary

During development, many animals choose between mutually exclusive fates, such as workers, soldiers, or queens in bees or ants. The choice between states is uniform throughout the animal since mixtures of these fates are not observed in the wild. The nematode *Caenorhabditis elegans* larvae integrate environmental conditions and have two choices: mature into reproductive adults or arrest development as dauer larvae—a latent form that can survive harsh conditions. The decision between both fates is governed by the hormone dafachronic acid (DA), however its regulation during development in response to environmental conditions has been unclear. In this study we show how two mechanisms are responsible for the integration of environmental conditions and the coordination of the decision between many tissues. We first show that a threshold mechanism integrates population density with the internal amount of DA made in the head. A normal population density has a low threshold of DA needed for worms to become adults, whereas a high population density increases this threshold and leads worms to develop into dauer larvae. We then show that the low levels of DA released from the head are amplified in the hypodermis (the main body syncytial epithelium) via a positive feedback loop, coordinating the decision over the animal. Disruption of this positive feedback yields abnormal adults. We propose that the positive feedback serves as a fate-locking mechanism enforcing an organismal binary decision—either adult or dauer—despite noisy and uncertain environmental conditions.

## Introduction

During development, organisms often face unpredictable and unfavorable environmental conditions that may decrease their fitness. In some cases, organisms of the same genotype develop into alternate phenotypes, each better adapted to a particular environment. Alternative phenotypes entail changes in metabolism, developmental programs, behavior, or morphology [1]. To predict the capacity of the environment to provide for reproductive development, animals integrate external environmental conditions, the internal state of nutrient supplies, and other variables [2]. In many cases, the integration culminates in a decision between two mutually exclusive alternative phenotypes. Therefore, robust developmental mechanisms have evolved to ensure that the animals coordinate development exclusively into only a unified phenotype, as uncoordinated development will be detrimental [3].

Organism-wide binary decisions are common throughout the animal kingdom and include examples such as sex determination, changes in coloration as a function of season, and caste differentiation in insects [3]. However, our knowledge of the mechanisms that regulate the decision between alternative phenotypes and coordinate the outcome across a multi-cellular organism is fragmentary, drawing on principles derived from studies of different model organisms [4]. Insects coordinate the fate of an alternative phenotype by altering hormone amounts above or below a threshold, during a hormone sensitive period, prior to metamorphosis [2]. A threshold distinguishes the two alternatives, yet it remains unclear how the thresholding mechanism is regulated and how the amounts of hormone are maintained throughout the body post-decision.

Transcriptional amplification mechanisms such as positive feedback loops have been shown to lock in binary fate decisions in phage [5], bacteria [6], and yeast [7]. A hallmark of such positive feedback mechanisms is that signals from a noisy environment can be forced into a bi-stable response by a threshold. Signal levels above the threshold will be amplified and maintained at a high abundance, acting as a memory module for a decision, thus enforcing a cell-specific fate. We sought to understand if such principles can be extended to hormonal regulation in multi-cellular organisms, specifically as a means to threshold, coordinate, and maintain the alternative phenotype in the selective environment.

To understand the interaction between genetic and hormonal regulatory mechanisms that integrate environmental conditions and coordinate a discrete developmental fate, we looked at the dauer decision in the free-living nematode *C. elegans*. During this decision, *C. elegans* integrates environmental conditions and chooses between the mutually exclusive fates, dauer or reproductive development. In favorable environments—plentiful food, moderate temperatures, and low population density—*C. elegans* develops rapidly through four larval stages (L1–L4) separated by molts, into a sexually reproductive adult. In unfavorable environments—high population density (indicated by high levels of a constitutively secreted dauer pheromone) limiting food or high temperature—animals can decide to develop into an alternative third larval stage, the dauer diapause, a developmentally arrested, long-lived form geared towards survival [8]. Dauer larvae do not feed and can endure harsh conditions, including starvation, desiccation, heat, and oxidative stress [8]. Accordingly, dauer larvae have profound morphological changes including an assault-resistant cuticle, pharyngeal constriction, and sealing of body cavities. Whereas adult nematodes live for about 3 wk, dauer larvae can survive several months. When returned to favorable conditions, dauer larvae resume development, molting into L4 larvae and adults [9]–[11]. In either reproductive or dauer mode, it is essential that the execution of the decision will be robust and that no mosaic phenotypes arise, as this will compromise survival. Moreover, understanding the decision-making process of diapause entry in *C. elegans* can illuminate analogous processes in parasitic nematodes whose infective stages are like dauer larvae and are regulated by some of the same signaling pathways [12]. A deeper understanding of the decision to become an infective juvenile as well as exit from this stage will facilitate the design of therapeutics that inhibit parasitic infection.

Although the major signaling pathways regulating dauer formation have been identified, the cellular and molecular basis of this binary decision is not clear. Environmental cues are detected by multiple sensory neurons that integrate inputs into hormonal outputs by unknown means [13]–[16]. Molecular analysis has revealed at least four signaling pathways. Components of neurosensory structure and guanylyl cyclase signaling are involved in sensing temperature, nutrients, and dauer pheromone [17], which regulate secretion of insulin/insulin-like growth factor and TGFβ peptides. Insulin and TGFβ signaling converge on a steroid hormone pathway, which metabolizes dietary cholesterol into several bile acid-like steroids, called the dafachronic acids (DAs) [18]–[23]. DAs serve as hormonal ligands for the nuclear hormone receptor transcription factor DAF-12, which regulates the life cycle fate decision. Liganded DAF-12 promotes reproductive development, whereas unliganded DAF-12, together with the co-repressor DIN-1S, directs the dauer fate. Thus, DAF-12 serves as a DA-responsive switch that determines whether an animal will undergo reproductive or dauer development [20]–[27]. The regulation of DAs during development as a function of environmental conditions remains largely unknown.

Here we identify the times of integration and commitment to this life cycle fate choice and show that environmental conditions affect the threshold at which discrete levels of DA bypass the dauer fate. Higher amounts of DA are necessary to implement and coordinate the reproductive decision throughout the whole animal. We show that a positive feedback loop, which amplifies the amounts of DA in the hypodermis (hyp7; WBbt:0005734), is required under some circumstances to produce the higher amounts of DA, while a negative feedback loop keeps hormones with normal bounds. Finally, we demonstrate that the amplification of DA in the hypodermis is responsible for the irreversibility of the decision and the proper execution of reproductive programs. We propose that hypodermal amplification of a hormonal signal acts as a commitment mechanism that enforces the binary decision.

## Results

### Points of Commitment during Life Cycle Fate Decisions

**Figure 1:**
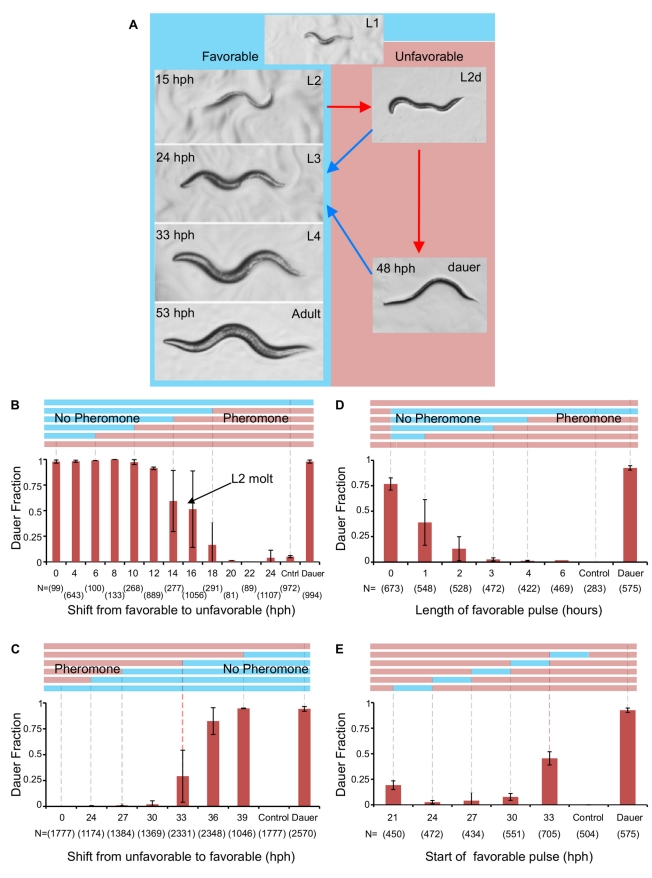
Commitment to dauer or reproductive development. (A) Developmental molt times of *C. elegans* N2 strain growing in favorable (blue) or unfavorable (red) conditions. (B–E) Time courses of commitment as a function of environmental conditions (pheromone). Top, representative colored bars indicate shifts to unfavorable conditions (red) or favorable conditions (blue). Bottom, means of dauer frequencies between biological replicates ± standard deviation. Numbers in parentheses indicate total worms per time point. (B) Period of pheromone sensitivity during L1 and L2: worms respond to pheromone between 12 and 18 hph. (C) Point of commitment to dauer: worms commit to dauer 33 hph denoted by the red dashed gridline. (D) Point of commitment from L2d to L3: worms commit to L3 after a 3 h pulse in favorable conditions when shifted at 24 hph. (E) Start time of pulse shifts to favorable conditions during L2d. Pheromone was added to worms 3 h post-shift to favorable conditions. Cultures shifted to favorable conditions at 33 hph show a higher ratio of dauers since worms commit to dauer at 33 hph (red dashed gridline). Control, worms grown without pheromone. Dauer, worms grown in 3% (v/v) pheromone with no shifts.

The decision between dauer arrest and reproductive growth is made at two points during early larval development. During late L1, worms develop into the L2 stage in favorable environments or into the dauer-capable pre-dauer (L2d) stage in unfavorable environments. During the mid-L2d stage, worms commit to either the dauer or resume reproductive development as L3 larvae (Figure 1A) [10],[28]. Golden and Riddle (1984) [28] showed that worms must be exposed to pheromone before the L1 molt in order to develop into the L2d stage and commit to the dauer fate before the mid-L2d stage. We re-visited these experiments and modified them to liquid culture to increase scale, homogeneity, and throughput. We measured the frequency of dauer formation in response to dauer pheromone while grown in the presence of sufficient food for adult development. Mean frequencies of life stages in favorable and unfavorable growth conditions were tightly distributed and highly reproducible, indicating the homogeneity of the liquid culture conditions (Figure S1A–B).

To identify when worms commit to reproductive development as L2 larvae, we performed a “shift-to-unfavorable” experiment by adding a high concentration of pheromone to synchronously hatched worms at progressive times. Worms stopped responding to pheromone at 18–20 hours post-hatch (hph; Figure 1B; 16.6%±21.4% dauer formation), which coincides with the beginning of the L2 stage. After this time, animals initiated reproductive development despite exposure to unfavorable conditions. To identify when worms commit to the dauer fate, we performed a “shift-to-favorable” experiment by growing synchronously hatched worms in unfavorable conditions (high concentration of pheromone) and washing away pheromone at progressive times. L2d worms committed to dauer during mid-L2d at 33 hph (Figure 1C; 29.1%±25.1% dauer formation), 18 h after the L1/L2d molt. Shifting worms to favorable conditions after this time did not affect their propensity to become dauers. We next identified when L2d worms commit to L3. We reasoned that L2d worms exposed to favorable conditions for longer times would have a higher propensity to develop into adults. The “shift-to-favorable” experiment was modified by growing worms under unfavorable conditions to obtain L2d animals, followed by a shift to favorable conditions at 24 hph. Worms were then returned to unfavorable conditions after varying amounts of time. We found that a 3 h pulse into favorable conditions was sufficient to commit L2d animals to reproductive development (Figure 1D; 0.02% dauer formation). Pulses at different start times during L2d had similar responses (Figures 1E, S1D). The L2d stage is thus divided into two periods: integration (from the beginning to the middle of the L2d stage, 16–33 hph) and commitment and implementation of the dauer program (33–48 hph).

### Two Thresholds of DA Are Required for Commitment to Adulthood and for Normal Adult Development

**Figure 2:**
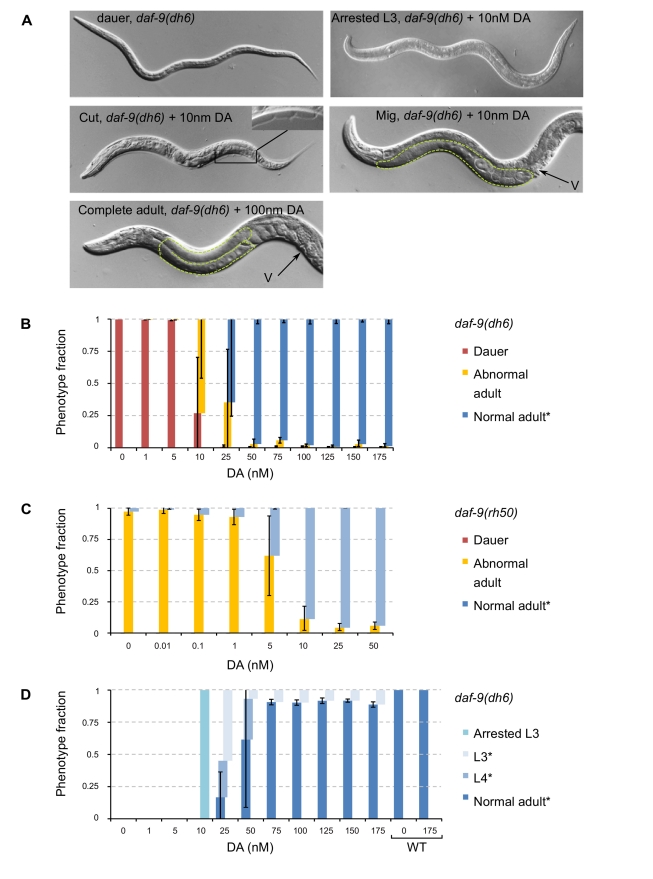
High amounts of DA are required for normal adult development. (A) Images of dauer, arrested L3, abnormal development Mig and Cut worms, and normal adults. Yellow hatched area encloses the gonad, v, vulva. (B) Distribution of developmental stages as a function of DA, scored 48 hph. Means of dauer (red), abnormal development (arrested L3, Mig and Cut; yellow) and normal adult (L3, L4, and young adult; blue) phenotype in *daf-9(dh6)* worms. (C) Distribution of developmental stages in the *daf-9(rh50)* background. (D) Distribution of stages in the adult fraction of phenotypes. Means of population proportions of stages indicate the relative developmental rate at each concentration of DA scored at 48 hph. Error bars represent means ± standard deviations across three biological replicates, *N*>500. Mig, gonad migratory defective; Cut, cuticle defective; YA; young adult. * Worms were gravid the next day.

We sought to find cellular and molecular candidates that could account for the dauer and L3 commitments. The two species of the DAs, Δ4-DA and Δ7-DA, are good candidates because synthetic DAs can fully rescue the Daf-c phenotypes of the null allele *daf-9(dh6)* (WBGene00000905) as well as *daf-7*/TGFβ (WBGene00000903) and *daf-2*/InsR (WBGene00000898) mutants [21]. Partial reduction of *daf-9* function results in animals that bypass the dauer stage yet exhibit abnormal gonadal morphogenesis and migration (Mig; WBPhenotype:0000594) and occasionally aberrant cuticle shedding (Cut; WBPhenotype:0000077) defects (Figure 2A) [18],[19]. Exogenous DA can also rescue these phenotypes [20],[21],[23],[24]. We thus hypothesized that a low amount of DA is required to bypass dauer and commit to L3, whereas a high amount is required for normal development.

To understand the physiological response to DA dose, dauer-constitutive *daf-9* loss-of-function mutants were treated with increasing amounts of Δ7-DA and measured for dauer and reproductive adult fates. Most *daf-9(dh6)* null animals developed into abnormal adults when supplemented with a minimum of 10 nM DA (Figure 2B, 74%±42% non-dauers), suggesting that a threshold of DA has to be crossed before committing to adult fate (dauer bypass DA threshold). Increasing the levels to 25 nM DA decreased the frequency of dauers to 99%±1% with about 66% of animals developing normally (Figure 2B). Further increase of DA to 50 nM increased the frequency of normal adults (Figure 2B). For a distribution of Mig and Cut phenotypes, see . Similar results were observed with animals homozygous for *daf-9(e1406)* or *daf-9(m540)* (Figure S2; worms were not synchronously hatched), both of which are strong loss-of-function alleles. By contrast, the weak loss-of-function allele *daf-9(rh50)* does not result in Daf-c phenotypes, but in highly penetrant Mig defects (95%±3%) [18]. In these animals, only 10 nM of DA was required to rescue over 90% of the Mig phenotypes (Figure 2C), revealing a 5-fold decrease in the amount of exogenous DA required to promote normal development compared to the stronger *daf-9* mutants (*dh6*, *e1406*, and *m540*; Figure 2S). Thus, *daf-9(rh50)* animals produce sufficient amounts of DA to bypass dauer development but require additional DA to develop into normal adults, consistent with our finding that different levels of DA are required for the two processes.

Many Daf-c mutants have a slower developmental rate, but the basis of this is not well understood (A. A., unpublished observations). To test the effects of DA on developmental rate, *daf-9(dh6)* worms were synchronously hatched in different concentrations of DA and scored for developmental stage at 48 hph (the time at which wild-type worms grown in favorable conditions are young adults (YAs) and worms grown in unfavorable conditions are dauers; Figure 1A) and for egg production the following day. At 25–50 nM DA, worms developed into L4, whereas worms supplemented with 75–175 nM DA already developed into YAs. Worms that were in the L4 or YA stages at 48 hph were gravid the next day. Exogenous addition of DA had no effect on wild-type growth rates (Figure 2D). These trends indicate that increases of DA levels can accelerate growth rate until it matches that of wild-type worms (Figure 2D).

### Pheromone Levels Regulate the Dauer Bypass DA Threshold and Reproductive Development

DA and dauer pheromone have opposite effects on dauer formation, with DA preventing and dauer pheromone promoting the dauer stage. We investigated the dose-response relationship when administered together, with respect to bypass of the dauer diapause and normal reproductive development. We hypothesized that the same dose of DA would be required to overcome dauer induced by pheromone as that seen in *daf-9(dh6)* null mutants.

**Figure 3:**
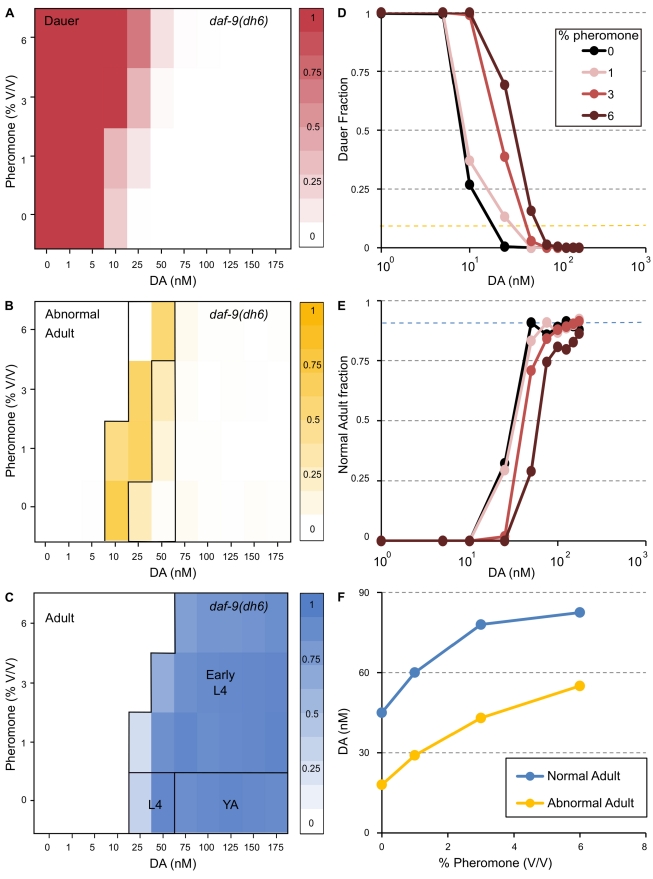
Dauer pheromone regulates the threshold for reproductive development. (A–C) Distribution of the phenotypes of the strain *daf-9(dh6)* when supplemented with DA: dauers (A), abnormal adults (B), and normal adults (C), as a function of DA and pheromone. Each pixel on the heat map is the mean fraction of population (see bar on the right for quantification) developing in the specific category, *N*>300 per pixel. Partition of abnormal adults and adults into sub-categories is detailed in Figure S3. (D) Concentrations of DA required to surpass the dauer bypass DA threshold as a function of pheromone. Yellow dashed line indicates 90% non-dauers in the population. (E) Concentrations of DA required for normal adult development without any arrested L3, Mig, or Cut phenotypes. Blue dashed line indicates 90% normal adults in the population. (F) Regression analysis of panels D and E; points correspond to 90% non-dauer (Yellow, 3D) and 90% normal adult (Blue, 3E, see Experimental Procedures for details of regression). Normal adult development requires an additional 30 nM DA above the amount for dauer bypass. Mig, gonad migratory defective; YA, young adult.

Synchronized populations of *daf-9(dh6)* worms were supplemented with a combination of DA and pheromone at different concentrations and scored for dauer, abnormal, and normal adult development at 48 hph (Figure 3A–C). Unexpectedly, addition of pheromone at 1%, 3%, or 6% (which induce 47%±4%, 92%±2%, and 95%±2% dauer in wild-type worms, respectively; Figure S1D) increased the concentration of DA necessary to exceed the dauer bypass DA threshold to 30, 45, and 58 nM, respectively (Figure 3F). Moreover, 90% of the population developed into normal adults if worms were supplemented with 30 nM of DA more than the amount required to bypass the dauer bypass DA threshold (Figure 3D–F), similar to the concentration of DA needed to bypass the dauer bypass DA threshold in *daf-9* worms without pheromone. These experiments demonstrate that dauer pheromone increases the amount of DA required to bypass the dauer bypass DA threshold and normal reproductive development.

### Dafachronic Acid Time of Action

**Figure 4:**
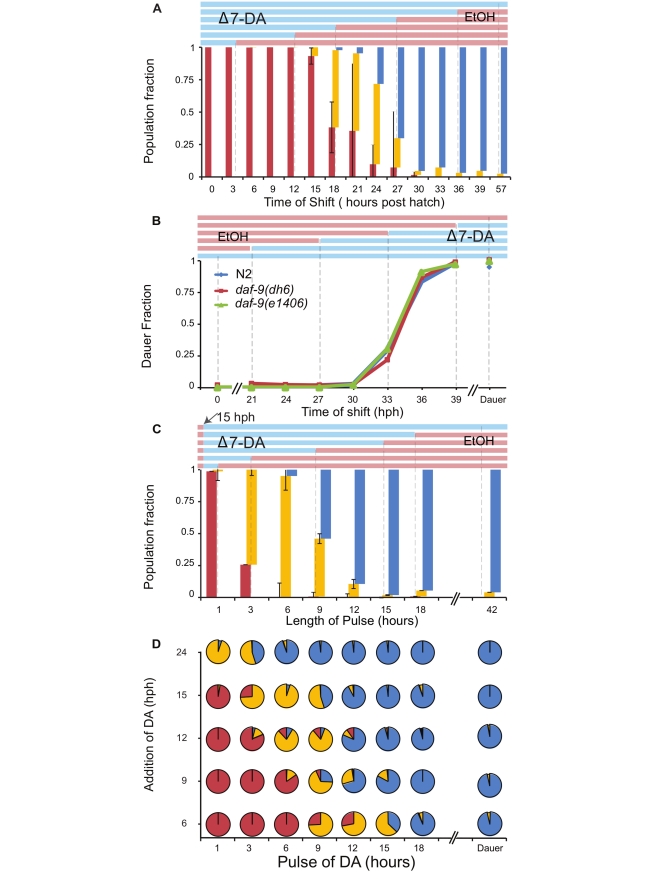
Timing requirements of Δ7-DA. Dauer, abnormal development and normal adult fates as a function of exposure times to 100 nM Δ7-DA. (A) *daf-9(dh6)* worms start responding to Δ7-DA at 15 hph and require an additional 12 h of Δ7-DA for normal adult development. Top, representative colored bars indicating the shift experiment: red bars indicate EtOH carrier and blue bars indicate Δ7-DA. Bottom, normal adult (blue), abnormal adult (yellow), and dauer (red) bars indicate the population fraction per time point. (B) Worms become refractory to Δ7-DA at 33 hph, the same time that they commit to dauer. N2 indicates worms shifted from unfavorable to favorable conditions as indicated in Figure 1C, and points indicate dauer proportions (abnormal development is considered non-dauer in this panel). (C) Pulses of Δ7-DA indicate the minimal time necessary for normal development when added at 15 hph. Top, length of pulses. Bottom, normal adult (blue), abnormal development (yellow), and dauer (red) bars indicate the population fraction per time point. (D) Worms have no memory of previous exposure to Δ7-DA before the L1/L2 molt. Pie charts indicate proportions of dauers (red), abnormal development (yellow), and normal adults (blue) as a function of total amount of time exposed to Δ7-DA (*x*-axis) when exposed to Δ7-DA at different hours post-hatch (*y*-axis). *N*>100 for all time points.

To understand the time of action of DAs and their role in life cycle fate decisions, we sought to identify three key points in the response to DA: (i) the time at which *daf-9(dh6)* animals start responding to DA to bypass dauer, (ii) the end of response to DA for the dauer decision, and (iii) the requirements of exposure to DA for normal development to maturity. Synchronously hatched *daf-9(dh6)* worms were shifted from media containing DA dissolved in EtOH to media containing EtOH alone (downshift) or vice versa (upshift). Analysis of downshift experiments revealed that worms started responding to DA after 15 hph, the same time that wild-type worms commit to L2 mediated reproductive development (Figure 4A). When DA was washed away before 15 hph, worms developed into dauers, despite previous exposures of DA indicating that previous exposures to DA had no effect on commitment to reproductive development. Removal of DA at time points after 15 hph prevented dauer formation to increasing extents, which could be divided into two phases: a minimum of 3 h on 100 nM DA during the responsive period was sufficient to prevent 61.7%±19.6% of the population from becoming dauers, but these animals developed as abnormal adults (Figure 4A, 15 to 18 hph), whereas an additional 12 h were necessary to drive 100% of the population to normal adult development (Figure 4A, 18 to 30 hph). To determine when *daf-9(dh6)* worms became refractory to DA, upshift experiments were performed during the L2d stage. Worms responded to DA until 33 hph, precisely at the same time that wild-type worms became refractory to pheromone (Figure 4B; correlation coefficient = 0.996). Next, we asked whether the total time exposed to DA or the specific time (stage) of exposure to DA were regulating the fate decision and development of normal adults. Pulse experiments revealed that worms committed to bypass dauer when exposed to DA at 15 hph or 24 hph for as little as 3 h (Figure 4C,D). When DA was supplemented for 3 h at 24 hph worms developed into normal adults (Figure 4D). These data correspond with addition of DA at 15 hph for 12 h, indicating that development into normal adults is a function of stage (27 hph, mid-L3) and a persistent exposure to DA (Figure 4D). Similar results were seen with the *daf-9(e1406)* allele (Figure S4). In sum, DA can affect the decision during a specific temporal window (15 to 33 hph) during the L2d stage, the same time that wild-type L2d worms integrate pheromone. Worms become committed to bypass dauer with a minimal exposure of 3 h in DA, but additional persistent exposure to DA over 12 h is necessary for normal adult development.

### Hypodermal *daf-9* Is Expressed as a Result of Reproductive Fate Decision

We wanted to understand how the spatiotemporal and tissue-specific regulation of *daf-9* is related to hormonal activity and stage commitments. The two bilaterally symmetric XXX cells (WBbt:0007855) express *daf-9* throughout all stages, suggesting that they may produce steady levels of DA [18],[19]. Hypodermal *daf-9* expression is more complex: hypodermal *daf-9* is weakly expressed in L3 larvae growing in favorable, low-stress conditions, strongly expressed in L3 larvae growing in mild stress conditions, and not expressed under high stress conditions that trigger dauer formation [20],[23].

First, we investigated the expression of *daf-9* mRNA during L2 and L2d in favorable and unfavorable conditions by whole animal qPCR in wild-type worms. Second, we examined the expression of DAF-9 protein levels and distribution with a translational DAF-9::GFP fusion by fluorescent microscopy (strain AA277; *lin-15(n765), dhIs64[daf-9::GFP, lin-15(+)]*; Gerisch et al., 2001 [18]; strain AA277 grows slower than N2 and therefore commitment to dauer occurs at 36 hph; Figure S5). We found that *daf-9* is regulated differently in favorable and unfavorable environmental conditions.

**Figure 5:**
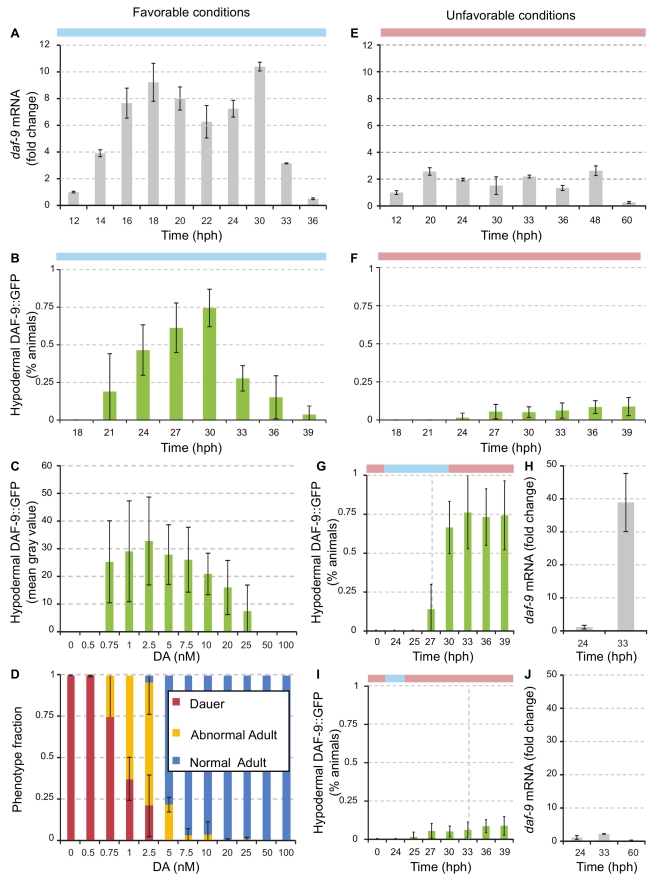
*daf-9* is transcriptionally upregulated in the hypodermis after commitment to reproductive development. (A,E) Fold change of total *daf-9* transcripts quantified by qPCR during development of synchronized broods (see Experimental Procedures, Table S2). Bars represent means ± standard deviations of fold change between biological replicates. (A) Development in favorable conditions. (E) Development in unfavorable conditions. (B,F) Fraction of worms expressing hypodermal *daf-9* during development in (B) favorable and (F) unfavorable conditions. (C,D) Δ7-DA regulates hypodermal *daf-9* transcription and development. *daf-9(dh6)* animals carrying the a *daf-9* promoter construct fused to *gfp* (*pdaf-9::gfp*) were grown on 0 to 100 nM DA. (C) Hypodermal GFP intensity. Animals grown in the presence of 0 to 0.5 DA show no hypodermal *daf-9* expression. Higher DA levels (0.75 to 10 nM DA) result in strong hypodermal expression, whereas higher levels (50 nM) abolish expression. Each bar represents the pixel intensity of a fixed area of the hypodermis of a single animal. (D) Phenotypic distribution of *daf-9(dh6)* worms expressing hypodermal *daf-9* when exposed to different concentrations of DA. Hypodermal upregulation is visible only at concentrations of DA that give rise to abnormal adults. (G,I) Hypodermal expression in worms grown in pheromone (red) and shifted to favorable conditions for a specified time window (blue) and shifted back to unfavorable conditions: (G) 6 h with a dauer frequency of 7%±5%, and (I) 1 h with a dauer frequency of 90%±10%. Bars represent the average of three biological replicates ± standard deviation. (H,J) Fold change of total *daf-9* transcripts quantified by qPCR when worms are shifted from unfavorable to favorable conditions (H), or maintained in unfavorable conditions (J).

In favorable conditions that promote reproductive development, total *daf-9* transcripts were upregulated 7±1.1-fold at 16 hph and peaked at 30 hph, with 10-fold upregulation (Figure 5A). All observed *daf-9* upregulation was due to the *daf-9a* isoform as we were unable to detect the *daf-9b* isoform (see Materials and Methods). Eighteen percent of worms started expressing hypodermal DAF-9::GFP at 21 hph, mid-L2 stage, reaching a maximum of 75%±12% at 30 hph, mid L3 (Figure 5B; *p*<0.0001). Presumably the delay between *daf-9* upregulation detected by qPCR to that observed by GFP is due to the translation of mRNA to protein and slower developmental rate of the AA277 strain.

Previous genetic experiments demonstrate that hypodermal *daf-9* expression is regulated by DAF-12 and hormone biosynthetic genes [20],[23]. To monitor *daf-9* transcriptional regulation in specific tissues as a function of DA, we performed experiments using a *pdaf-9::gfp* transcriptional promoter construct in the *daf-9(dh6)* background. This promoter construct largely recapitulates the behavior of the translational fusion, suggesting that the majority of regulation occurs at the level of transcription. At low DA concentration (0–0.5 nM), expression was seen only in the XXX cells, and all animals developed as dauers (Figure 5C–D). As DA concentration was increased to 0.75–7.5 nM, expression in the hypodermis dramatically increased by mid-L2, suggesting positive amplification (Figure 5C–D). Notably, within the range of 1–5 nM DA, animals bypassed dauer but exhibited the abnormal development phenotypes (Figure 5C,D). Hypodermal expression was decreased at 10 nM or shut off (50–100 nM), suggesting suppression of *daf-9* expression. At these higher concentrations (>10 nM) all animals developed into normal adults. Exogenous DA and hypodermal *daf-9* upregulation have an inverse relationship; intermediate and high levels of DA promote intermediate, and low levels of hypodermal *daf-9*, which correspond to states of abnormal and normal development, respectively.

In unfavorable conditions, total *daf-9* transcripts were not significantly upregulated in L2d animals committed to dauer (Figure 5E,J; *p* = 0.14). All observed *daf-9* upregulation was due to the *daf-9a* isoform as we were unable to detect the *daf-9b* isoform (see Materials and Methods). Nearly all worms grown in unfavorable conditions failed to show hypodermal DAF-9::GFP expression during L2d or dauer (Figure 5F; 92%–100%, *p* = 0.18). Shift-to-favorable experiments revealed a 40-fold upregulation of *daf-9* transcripts in wild-type worms committed to reproductive development (Figure 5H). When L2d worms were pulsed into favorable conditions at 24 hph for a 6-h window, 76%±12% showed hypodermal DAF-9::GFP expression with onset as early as 27 hph (Figure 5G). Hypodermal *daf-9* expression was retained even when worms were shifted back to unfavorable conditions. Conversely, 93%–99% of worms shifted to favorable conditions for 1 h did not express hypodermal DAF-9 (Figure 5I). These results correlate temporally with the minimum time that wild-type worms require a pulse in favorable conditions to bypass dauer and suggest that hypodermal *daf-9* expression could be a cause or consequence of a decision to develop into L3.

### XXX Cells Regulate Hypodermal *daf-9* Expression in a Shift from Unfavorable Conditions

**Figure 6:**
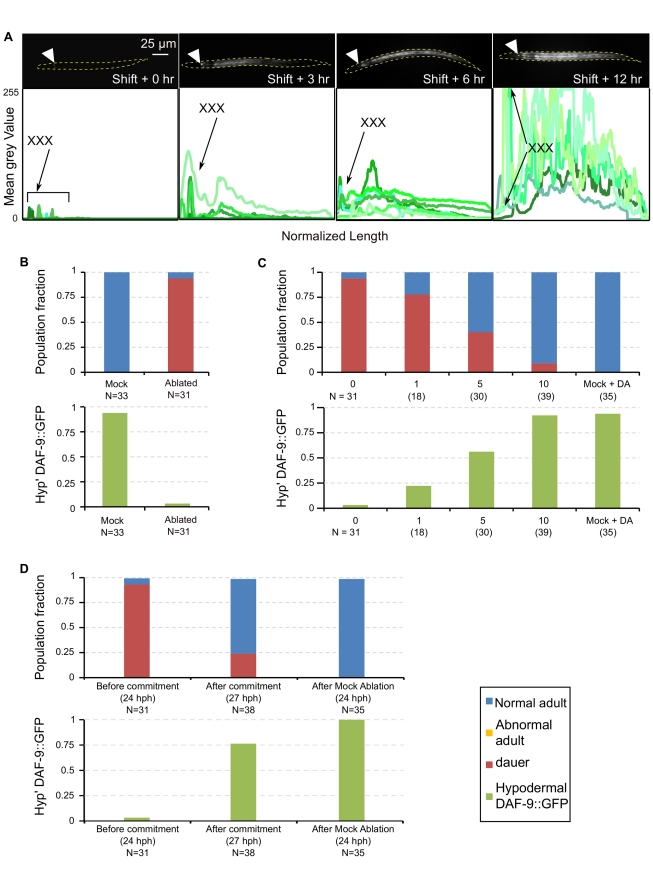
Hypodermal *daf-9* expression propagates from anterior to posterior upon commitment to the L3 fate. (A, top) Fluorescent images of worms at each time point are shown at shift from unfavorable to favorable at 0 (24 hph) h (leftmost image) through 12 hph (rightmost image). Arrowheads mark the XXX cells. (A, bottom) Expression of hypodermal *daf-9* was quantified along the anterior posterior axis in 4–6 worms in each time point. Each green shaded histogram represents the mean grey value of DAF-9::GFP per worm, normalized to length. Different worms were imaged at each time point (see Text S1 for details of analysis). (B–D) XXX cells and Δ7-DA are required to initiate hypodermal daf-9 expression and reproductive development. (B) Cells were ablated during L2d and recovered in favorable conditions. (C) L2d ablated worms were let to recover on 1, 5, or 10 nM Δ7-DA. All worms expressing hypodermal *daf-9* developed into normal adults with no Mig or Cut phenotypes. (D) Worms were grown to L2d, XXX cells were ablated after commitment to L3 at 27 hph. * *p*<1×10^−4^, ** *p*<1×10^−10^.

Worms committed to reproductive development had transcriptional upregulation of *daf-9* in the hypodermis, likely resulting in the production of the high levels of DA. We asked whether the XXX cells play a role in hypodermal *daf-9* upregulation. Notably, we observed that after a shift from unfavorable to favorable conditions, hypodermal DAF-9::GFP expression was observed in a spatiotemporal manner along the anterior posterior axis (Figure 6), first and most strongly in the head region before expression spread to more posterior regions. This led us to hypothesize that under these conditions, XXX cells (located at the anterior) might act as a source of DA, releasing a small amount that is amplified and propagated in the hypodermis from anterior to posterior.

To test this hypothesis, we removed the XXX cells with a laser microbeam. We ablated XXX cells in worms expressing a translational DAF-9::GFP fusion developing in high pheromone concentration at 24 hph (mid L2d, pre-commitment), and worms were allowed to recover in favorable conditions. Nearly all (30/31) XXX-ablated worms lacked hypodermal DAF-9::GFP expression and developed as dauers, while 29/31 control mock-ablated L2d animals developed into adults (Figure 6B; *p*<1×10^−10^, Figure S6A). Therefore, intact XXX cells are necessary for L2d larvae to respond to favorable conditions, committing to reproductive development and initiating hypodermal *daf-9* expression.

We next tested whether DA could rescue the dauer arrest caused by ablation of XXX cells. Worms were grown in high pheromone concentration and XXX cells were ablated at 24 hph (L2d before commitment), and shifted to growth in favorable conditions supplemented with 0, 1, 5, or 10 nM DA. An increasing frequency of both hypodermal DAF-9::GFP and normal adult development was observed as higher concentrations of DA were supplemented. Rescue with 1 nM DA yielded 22% adults and 78% dauers (*N* = 18), rescue with 5 nM DA yielded 56% adults and 44% dauers (*N* = 30), and rescue with 10 nM DA yielded 92% adults and 8% dauers (*N* = 39; Figures 6C and S6B). All XXX-ablated worms supplemented with exogenous DA developed either as normal adults or as dauers, with none of the Mig or Cut phenotypes seen in *daf-9(dh6)* worms at these concentrations of exogenous DA (Figure 2B). These results suggest that in the absence of the XXX cells, hypodermal *daf-9* upregulation can be induced with as little as 1 nM DA, resulting in normal adult development. By contrast, in the *daf-9* null background hypodermal *daf-9* amplification is not possible, leading to abnormal development at low DA levels.

To test whether XXX cells act as a source of DA later in development, we ablated XXX cells after commitment to L3. Ablation at this time had no effect and resulted in worms that expressed hypodermal DAF-9::GFP and matured to adulthood (Figure 6D; *p* = 2×10^−9^, Figure S6C). Therefore, XXX cells act as a source of DA during the dauer decision and become dispensable later.

## Discussion

Here we characterized a molecular mechanism connecting environmental signals to hormonal regulation during the commitment to reproductive development in *C. elegans*. We identified specific time windows during which worms integrate their environmental conditions and make a decision between reproductive development and diapause. We demonstrate how environmental conditions regulate the threshold of hormone required to commit to a certain fate. Finally, we find that environmental information is funneled to the neuroendocrine XXX cells, which upon a decision to commit to adulthood initiate activation of a positive feedback loop of hormonal production. This mechanism, consisting of a proposed gene regulatory network, regulates robust hormonal amplification, thus enforcing the fate decision throughout the organism.

### Critical Periods of Environmental Integration

**Figure 7:**
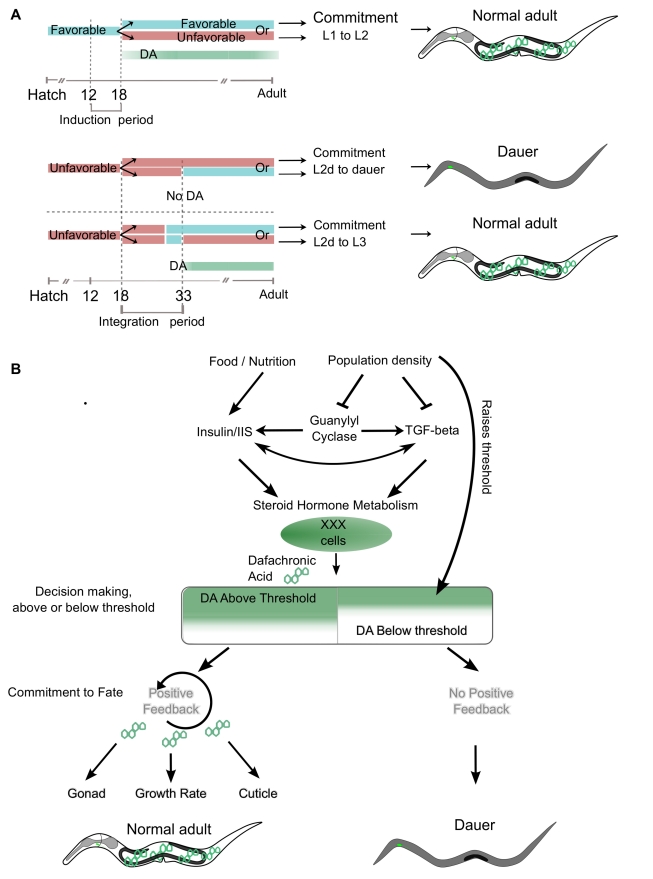
A feedback loop amplifies a DA signal leading to coordinate development. (A) Environmental conditions overlap with DA time of action. Top, growth in favorable conditions (blue) during the induction period commits worms to adulthood. Committed worms develop into adults even if shifted to unfavorable conditions (red). Commitment to adulthood correlates with the time of action of DA. Middle and bottom: worms grown in unfavorable conditions during the induction period develop into L2d worms which decide between regular development and alternative development during the integration period (between 15 and 33 hph, hashed grey lines). Worms commit to the dauer fate at 33 hph or to adult development if exposed to favorable conditions for 3 h. Development to dauer correlates with no DA production, and development to adulthood correlates with production of DA. (B) Noisy and uncertain environmental information is measured by sensory neurons and reduced in complexity into the four signaling pathways. Information complexity is reduced further to the XXX cells, the primary source of DA. If nascent amounts of DA produced by the XXX cells bypass the dauer DA threshold, worms will develop into reproductive adults, and if DA levels are under the threshold, worms will develop into dauers. Upon commitment to reproductive development, DA originating from the XXX cells will initiate the hypodermal *daf-9* positive feedback loop, thus increasing the amounts of DA and thus locking the adult decision and producing sufficient amounts of DA for complete adult development. The positive feedback loop canalizes development guaranteeing that sufficient amounts of DA are produced so that abnormal phenotypes are not expressed in adult worms.

We analyzed the commitment of developing *C. elegans* larvae to reproduction (L3 larvae) or delayed reproduction (dauer larvae) in liquid culture, enabling a large and highly synchronized brood, amenable to facile and reproducible changes in environmental conditions. We showed that larvae exposed to favorable conditions for the first 12–18 h no longer entered dauer arrest when subjected to later pheromone exposure (Figure 7A). Conversely, if grown in unfavorable conditions between 12 and 18 hph, worms were induced to the pre-dauer stage, L2d. Worms integrate environmental conditions during the L2d stage and become irreversibly committed to the dauer fate by 33 hph. However, L2d worms can commit to the reproductive fate if pulsed with favorable conditions for 3 h before the 33 hph commitment point is crossed (Figure 7A). Our identification of the times at which these life cycle fate decisions occur allowed us to couple changes in the environment to the known molecular and cellular components involved in this decision.

### Environmental Conditions Regulate the Hormonal Threshold for Commitment

Timing of the decision period is congruent with the requirement for the hormone DA and the expression of the DA biosynthetic gene *daf-9*. In this view, favorable conditions equate with the presence of DA, while unfavorable conditions equate with its absence. Starting from 15 hph, pulses of DA 3 h or longer will bypass dauer with no memory to previous exposures to DA. Similarly, *daf-9* mutants stop responding to DA at 33 hph, mid-L2d stage. Thus, these periods of DA sensitivity overlap with the response to changes of population density in the environment.

Our studies suggest that two thresholds of DA must be crossed to ensure proper reproductive development: DA levels above the dauer DA bypass threshold will specify reproductive development, and DA levels below the normal adult threshold will specify dauers. If worms produce DA levels between the two thresholds, they will develop into abnormal adults. Importantly, the two thresholds only become apparent in *daf-9* mutants, which uncouple the dauer bypass threshold from the normal adult threshold. Addition of DA to *daf-9* mutants indicates that 10 nM DA is sufficient to overcome the dauer DA bypass threshold in liquid culture and 1–5 nM are sufficient on plates, therefore committing worms to L3 development. These animals require an additional 30 nM DA to promote normal gonadogenesis and cuticle formation, thus developing into normal adults. Higher levels of DA increase developmental rate. In addition, dauer pheromone can raise both DA thresholds, thus increasing the fraction of dauers in a population. Therefore, worms that produce DA levels above the dauer DA bypass threshold, or lower the threshold itself, will develop into adults. The difference of 30 nM between both thresholds is constant regardless of the amount of pheromone to which worms are exposed. These observations suggest that pheromone has additional targets downstream or parallel to DA production that antagonize reproductive development (Figure 7B).

### Spatiotemporal and Genetic Regulation of the Dauer Decision

What might be the molecular and cellular correlates of these two thresholds? The cytochrome P450 DAF-9 is limiting for DA production. *daf-9* is expressed in the XXX cells from hatch and throughout development, and in the hypodermis starting from mid-L2 until L4. The timing requirements for DA described above suggest that the commitment to adult development through the L2 stage is made early in L2 between 15 and 18 hph, a time that precedes visible hypodermal *daf-9* expression.

At high population density, the XXX cell appears to be source of DA required for the dauer decision, and the hypodermis amplifies DA production leading to normal development. When worms are shifted from unfavorable to favorable conditions, the dauer bypass DA threshold is lowered for a sufficient amount of time and the XXX cells presumably make a sufficient amount of DA to pass that threshold. Once the XXX cells release a small amount of DA, the hypodermis amplifies this signal leading to normal adult development. This amplification is visible as anterior to posterior propagation of hypodermal *daf-9* expression originating in proximity of the XXX cells. If the XXX cells are ablated, there is no source of DA to trigger hypodermal *daf-9* transcription and animals develop as dauer larvae. Hypodermal *daf-9* amplification is triggered if XXX-ablated animals are supplemented with as little as 1 nM DA. Lastly, the onset of hypodermal *daf-9* upregulation renders worms insensitive to removal of XXX, thus conferring the irreversibility of the decision and committing worms to the reproductive fate (Figure 7A).

In favorable conditions, the XXX cells and the hypodermis may share responsibilities. Under these conditions *daf-9* expression in the XXX cells appears steady and hypodermal expression low. The XXX cells are sufficient but not necessary for committing to reproductive fate: rescue of the *daf-9(dh6)* putative null by a XXX cell-specific DAF-9 construct leads to adult development. Ablation of the XXX cells during the L1 stage, in worms grown in favorable conditions, results in 30% of animals developing as dauers [29]. However, the hypodermis can overcome this deficiency of XXX signaling by *daf-9* upregulation [18],. Hypodermal expression of *daf-9* works as a homeostatic regulator since low amounts of DA increase transcription of *daf-9* in a *daf-12*-dependent manner, whereas sufficient production of DA by the XXX cells is not followed by hypodermal upregulation of *daf-9* during the L2 and L3 stages.

From the *daf-9* expression pattern, we infer that in favorable conditions DA is released in low levels over a long period of time, whereas worms developing in unfavorable conditions to adulthood release a burst of DA over a short period of time. This also implies that worms have a mechanism of counting and integrating hormone levels to reach the threshold of the dauer decision (Figure 7B). We speculate that this could be achieved by various levels of DA swapping DAF-12/DIN-1 or other co-repressor complexes for DAF-12/co-activator complexes, a known mechanism in nuclear receptor signal transduction [30].

Consistent with the importance of the XXX cells to the dauer decision, many components of the dauer regulatory pathways are expressed in these cells, including *ncr-1*, the Niemann-Pick C1 homolog, *hsd-1* encoding a 3β-hydroxysteroid dehydrogenase, and *sdf-9* and *eak-6*, which encode tyrosine phosphatases, *eak-3*, *eak-4*, and *eak-7*, novel proteins localized to the plasma membrane [18],[19],[27],[29],[31],[32]. These components as well as others could regulate enzymatic activities, availability, or hormone transport to and from the XXX. Additional activities in the dauer pathways could regulate the amount of DA produced in the XXX cells and the adult DA threshold, in endocrine or target tissues.

### A Model for Reproductive Commitment

The spatiotemporal and homeostatic regulation of *daf-9* provides a molecular mechanism that can explain the phases in the decision between dauer and reproductive development: integration of environmental and internal information, signaling of the decision, and implementation of a coordinated and irreversible decision. We propose that integration occurs by a process of information reduction and that signaling and implementation occur by a process of amplification and propagation upon reception of a discrete signal (Figure 7B). Complex information from the environment is measured by at least six neuron pairs (ASI, ADF, ASG, ASJ, ASE, and ASK [13],[15],[16]) and reduced in complexity by the Insulin/IGF, TGFβ [33], guanylyl cyclase [17], and steroid hormone pathways [18]–[21],[23],[24]. We propose that the XXX cells then integrate information from these signal transduction pathways and commit to reproductive development by releasing DA (Figure 7B). The latter phases, signaling and implementation of the decision, involve amplification and distribution of the discrete signal via a positive feedback loop in the hypodermis, thus ensuring a coordinated response over the whole animal. Amplification of the signal leads to independence from the integration apparatus. Should environmental conditions change and XXX cells cease to release DA, the amplification and diffusion over the whole body guarantees that all tissues will receive the necessary amounts of DAs required for normal adult development, thereby preventing inappropriate development in any cell lineage. In the future, it will be important to dissect further components of these life cycle commitments as well as the upstream mechanisms that weigh the decision within the XXX cells.

### Comparison with Other Hormonal Regulation in Other Organisms

Although there are clear differences across taxa, hormonal regulation in *C. elegans* bears many similarities to insect and mammalian hormonal regulatory mechanisms. First, external cues such as nutrients and photoperiod as well as internal cues such as body size or organ development affect developmental progression. Second, the hormone sensitive period overlaps with the environmental sensitive period and acts as a cue integrator also observed in the insects lepidoptera, hymenoptera, and diptera [34]. Third, developmental progression and coordination of development are relayed via the insulin/IIS and TGFβ/activin signal transduction pathways. In particular, the insulin/IIS pathway positively regulates reproductive growth in *C. elegans* [35],[36], insects [37], and mammals [38],[39], and may do so by converging on steroidogenic pathways [20],[23],[37],[40]. Conversely, a reduction of Insulin/IGF signaling increases the propensity for diapause in nematodes [4], and insects [37], as well as torpor in mammals [41],[42]. TGFβ/activin signaling also controls steroidogenic enzymes and influences mammalian reproductive development [43]. Fourth, insect TGFβ/activin signaling regulates metamorphosis by controlling expression of a subset of steroidogenic enzymes through Insulin/IGF signaling and the prothoracicotropic hormone PTTH. PTTH is an insect peptide hormone that regulates developmental timing and body size at metamorphosis [44]. Its release from prothoracicotropic neurons and binding to its cognate receptor Torso in the prothoracic gland results in stimulation of steroidogenic gene expression and the production of Ecdysone [45]. In mammals, pulses of gonadotropin-releasing hormone (GnRH) may work analogously to PTTH to signal commitment to reproduction [2],[38]. Although *C. elegans* lacks PTTH or GnRH hormones, conceivably other neuropeptides could take on this role. Fifth, the discrete spatial regulation of primary and secondary sources of steroid metabolism resembles somewhat those in insects, in which the precursor Ecdysone is produced in the PG, but the final product 20-hydroxyecdysone is converted in the peripheral tissues including the epidermis, midgut copper cells, Malpighian tubes and the fat body [44]. Last, hymenopterans and coleopterans have been shown to regulate alternative phenotypes by modulating the hormonal threshold via secreted pheromones [2]. The ant *Pheidole bicarinata* regulates the threshold of Juvenile Hormone (JH), causing the differentiation between worker and soldier ants. Worker ants are determined by a sub-threshold dose of JH, while soldier ants are determined by above threshold amounts of JH. Ants that have committed to the soldier caste will secrete a soldier inhibiting pheromone, which raises the JH threshold in pre-committed ants [46].

We have demonstrated that a simple network architecture of positive feedback can both lock in a fate decision and convey irreversibility of a decision. Because the hormonal regulatory mechanisms found in the worm are similar to insect and mammalian systems, the relative simplicity of the *C. elegans* may prove beneficial in elucidating the environmental, cellular, and molecular mechanisms of decision-making involved in reproductive commitments in multi-cellular organisms. It will be particularly interesting to determine if the commitment role of hormonal amplification and feedback plays an analogous role in other animals as observed in *C. elegans*.

## Materials and Methods

### Growth Conditions

All worms were handled using standard growth and cultivation techniques using the bacterial strains HB101 and OP50 as food sources [47]. Unless otherwise stated all liquid cultures were grown in glass flasks at ∼1 worm per µl at 20°C in S complete medium supplemented with 7.5 mg/ml HB101 as described in [47] in an Innova 4230 incubator at 180 RPM. The wild-type strain used was N2 (Bristol).

### Synchronous Hatching of Large Broods

Worms were hatched synchronously essentially as described by [48]; changes are described in the SOM.

### Pheromone Assays

Crude pheromone was prepared as described in [28]. Each pheromone extract was tested on N2 worms (1 worm per µl) and diluted so that 3% (v/v) would yield 90%±2% dauer arrest in a culture supplemented with 7.5 mg/ml of HB101.

### Shift Assays

Synchronous broods were grown as described above to the L2d stage by supplementing media with 3% (v/v) pheromone, partitioned into multiple parallel cultures, and grown in glass tubes. Shift to favorable: at specified times, broods were washed 3 times in S basal to remove pheromone. Cultures were re-suspended in S complete medium containing HB101 and calibrated for density. Shift to unfavorable: broods were supplemented with 3% (v/v) pheromone and grown in glass tubes. At specified time points (L2d), worms were partitioned into a control sample and experimental samples, which were washed 3 times with S basal. Worms were suspended in S complete medium and allowed to grow for specific time periods until 3% pheromone (v/v) was added.

### Dafachronic Acid Assays

Liquid culture: Δ7-DA was solubilized in 100% EtOH to necessary concentrations. Liquid culture assays were performed by adding EtOH-solubilized Δ7-DA in S basal medium. NG agar plate assays were performed by resuspending EtOH-solubilized Δ7-DA in S basal with OP50 and spreading on plates. Worms were picked onto Petri plates not more than 1 d after Δ7-DA was added to those plates. For the p*daf-9*::gfp experiment Δ7-DA was added on 3 cm NG agar plates, seeded with OP50.

### Scoring Abnormal Development

*daf-9(dh6)*, *daf-9(e1406)*, and *daf-9(m540)* worms were grown in liquid culture with different concentrations of Δ7-DA as described above. Worms were washed once with S basal medium to remove HB101 and mixed with S basal medium containing 1 mM sodium azide (to limit worm movement), spotted onto a 24-well plate. Worms were scored for gonad migration and cuticle shedding. Phenotype frequencies were calculated as the means of three biological replicates ± standard deviation.

### Regression Analysis of Dauer Bypass DA Threshold

Plots of *daf-9(dh6)* supplemented with 0%, 1%, 3%, and 6% pheromone as a function of DA were fit to a sigmoidal curve of the form f(x) = 1/(1+x^n^), where x is a log transformed concentration of DA, and n is the hill coefficient of the slope. Each pheromone concentration hill coefficient and EC50 were used to solve the EC90; the concentration of DA to bypass 90% dauer formation or 90% normal adult formation (Figure 3F, yellow and blue curves) according to the equation: EC90 = (90/(100-90))^1/n^ * EC 50.

### Commitment to Adult or Dauer

Frequencies were calculated within each biological replicate and means of frequencies ± standard deviation were calculated between biological replicates. We determined the point of commitment at the measurement times with highest standard deviation as it represents the tipping point of a transition between non-committed to committed worms. We calculated a q-statistic based on a Tukey type multiple comparison test for differences among variances (Zar 2009 [49]; Table S1).

### Homogeneity of Liquid Culture

Stage distributions were compared between three biological replicates in favorable and unfavorable conditions. A Bartlett's test [49] was used to determine if variances were significantly different between all stages of development.

### Significance of Hypodermal daf-9 Expression

Analysis was performed by a one-way ANOVA (Figure 5B,F). Significance of hypodermal upregulation in favorable versus unfavorable conditions after different time windows in favorable conditions was analyzed using a two-tailed *t* test between worms scored 30 hph (Figure 5G,I). Significance of transcriptional upregulation was analyzed by one-way ANOVA across all time points (Figure 5A) and paired *t* tests between L2d uncommitted to L2d committed to dauer and to L2d committed to L3 (Figure 5H,J).

### Cell Ablation

AA277 worms (*lin-15(n765)*, *dhIs64[daf-9::GFP, lin-15(+)]*) were grown to L2d stage in pheromone as described above. We found it necessary to use fluorescently labeled XXX cells as they migrate from the nose tip to the posterior region of the anterior bulb [50]. Worms were placed on glass slides with 5% agarose and 1 mM sodium azide in S basal, and laser microbeam ablations of the XXX cells were performed as described [51]. Worms were allowed to recover for 2 h before re-mounting on slides and verifying successful ablation by determination that no fluorescence signal was seen from either XXX cells. Worms were then transferred to either NG agar plates or NG agar plates supplemented with 1, 5, or 10 nM Δ7-DA. All ablations were coupled with mock-ablation controls. Statistical significance of observed differences between ablations and controls was determined using Fisher's exact test [49].

### Imaging

Strain AA277 was grown in liquid culture as described above. At specific times, worms were washed once in S basal medium and plated on glass slides with 5% agarose and 1 mM sodium azide in S basal. Worms were scored for hypodermal DAF-9::GFP under 40× magnification using a Zeiss Axiovert 200 microscope with a 200W mercury bulb.

Anterior posterior DAF-9::GFP expression: Each worm was imaged using both Nomarski and fluorescence using a CoolSnap HQ camera (Photometrics, Tucson, Arizona, USA) run through Metamorph software (MDS Analytical Technologies, Toronto, Ontario, Canada). Four to six worms were imaged per time period at 5 ms per Nomarski image and 400 ms per fluorescence image. Worms were straightened computationally, normalized to length, and mean grey value was quantified using custom software written in Matlab (see Text S1 for details).

### Hypodermal p*daf-9* Expression

Different concentrations of DA were added to NG agar plates, seeded with OP50. One day later, 10 reproductive *daf-9(dh6) pdaf-9::gfp* adults (grown in the presence of 250 nM DA) were placed on each plate for egg laying. F1 progeny were scored for hypodermal *daf-9* expression levels and dauer, molting, and gonadal cell migration phenotypes. Experiments were performed at 20°C and repeated at least twice. The GFP fluorescence was imaged through a Zeiss Axio Imager Z1 and photographed with an AxioCam MRm camera. Pixel intensity over a fixed area was measured with AxioVision 4.7 software.

### Worm Preparation for mRNA Analysis

Synchronous populations of worms were grown at 20°C either in favorable (2 worms per µl, 15 mg/ml HB101) or in unfavorable conditions (3% pheromone v/v, 2 worms per µl, 15 mg/ml HB101). At each time point, 104 worms were washed 3 times in S basal medium without cholesterol (pH = 6) to decrease bacterial load and to wash off excess pheromone. Samples were concentrated in 100 µl volume and suspended with 1 ml TRIzol reagent (Invitrogen, USA) and mixed with 0.6 µl/ml Linear Poly acrylamide, used as a carrier [52], flash frozen using liquid nitrogen, and stored at −80°C until processed.

### RNA Isolation

RNA purification using TRIzol was adapted from the manufacturer's protocol and is described in the SOM. RNA was subjected to quality control by Nanodrop spectrophotometry (A260/280 ratio) and Agilent Bioanalyser (S28 to S18 ratio). Samples were processed if the A260/280 ratio was above 1.9 and the S28 to S18 ratio was above 1.8. RNA was digested with RNase-free DNase (Ambion, Austin, Texas) according to the manufacturer's instructions. Total RNA was made into cDNA by reverse transcription reaction using Superscript III (Invitrogen, San Diego, California). mRNA was selected for reverse transcription by using oligo dT primers. Reactions containing no reverse transcriptase were carried out in parallel. cDNA was purified on silica columns (Qiagen, Venlo, Netherlands) and diluted to 16 ng/µl for subsequent qPCR analysis.

### qPCR Experimental Design and Analysis

*daf-9* transcripts were analyzed with three pairs of primers spanning different exons according to the WS190 gene model (http://ws190.wormbase.org). Each of the three amplicons was between 115 and 183 bp in length and included sequence from two exons (Table S2). All qPCR reactions were prepared using Roche SYBR Green I Master (Roche Diagnostics) and carried out in a Roche Lightcycler LC480. Data analysis was performed according to the ΔΔCt method [53]. Efficiency values of each primer set were empirically determined by performing a dilution series on pooled cDNA. Transcripts were analyzed if they crossed the Ct threshold before 34 cycles. Control genes were determined empirically by measuring gene expression that did not change significantly (Pearson correlation >0.995) during larval development (L1 through L4) and dauer fate. *daf-9* relative abundance was determined as follows: for mRNA processed from worms grown in favorable conditions, *daf-9* was normalized to the geometric mean of control genes *pmp-3* and- *Y45F10D.4* [54]. mRNA processed from worms grown in unfavorable conditions was normalized to relative abundance levels of *ver-2*, a gene expressed only in the ADL neurons [55]. *daf-9* fold change was determined by normalizing all time points to relative abundance in the L1 stage. Error bars represent mean fold change ± standard deviation across two technical replicates originating from three biological replicates (six data points).

### Accession Numbers

Accession numbers from http://www.wormbase.org: Genes: *daf-2*: WBGene00000898, *daf-7*: WBGene00000903, *daf-9*: WBGene00000905, *daf-12*: WBGene00000908, *daf-16*: WBGene00000912, *din-1*: WBGene00008549, ncr-1: WBGene00003561, *hsd-1*: WBGene00012394, *sdf-9*: WBGene00004748, *eak-3*: WBGene000022356, *eak-4*: WBGene00009955, *eak-6*: WBGene00008663, *eak-7*: WBGene00010671.

Phenotypes: Mig, WBPhenotype:0000594, Cut, WBPhenotype:0000077.

Cells: XXX: WBbt:0007855, hyp7: WBbt:0005734.

## Supporting Information

Figure S1 **Time course of staged animals grown in environmental conditions.** (A) Worms grown in favorable conditions of 7.5 mg/ml HB101 as a bacterial food source, ∼1 worm per µl and 20°C and (B) worms grown in the aforementioned conditions, supplemented with 3% v/v crude pheromone extract. Points indicate means ± standard deviation across three biological replicates. (C) SDS resistance begins 4–6 h after the L2d to dauer molt. Two time courses are indicated using pheromone extracts produced from different worm broods on different days. (D) Percent of dauers formed when pheromone extract was added into growth containing WT worms grown in conditions described above.
(TIF)

Figure S2 **High amounts of DA are required for complete adult development.** Distributions of dauer (transparent), Mig (dotted), Cut (hatched), and normal adult (grey) phenotypes when mutants were hatched after a non-synchronous bleach (worms typically hatch over a 15 h window at these growth conditions). (A) *daf-9(dh6)*, (B) *daf-9(e1406)*, (C) *daf-9(m540)*, and (D) *daf-9(rh50)*. Red represents all dauers, yellow represents all abnormal adult phenotypes, and blue represents all complete adults. Bars represent means ± standard deviations across three biological experiments. Numbers in parentheses indicate total worms counted per time point.
(TIF)

Figure S3 Distribution of developmental stage and phenotype of synchronously hatched *daf-9(dh6)* in combinations of dauer pheromone and Δ7-DA. (A–C) Color intensity on each pixel on the heat map represents the mean of three biological experiments. For example, the pixel 0% pheromone and 10 nM DA have a population composed of 28.5% dauer, 68.2% arrested L3, and 3.3% Mig. Red, regions dominated by the dauer phenotype. Yellow, areas dominated by the incomplete adult or arrested L3 phenotypes. Blue, areas dominated by the adult phenotypes: early L4, L4, and young adult. Besides the dauer and the arrested L3 stages, all other stages were gravid adults the following day. (D,E) DA controls developmental rate: each pixel is an index composed of a weighted sum of developmental distribution normalized to the maximal growth rate of WT N2 worms.
(TIF)

Figure S4 Temporal activity of Δ7-DA. This provides similar experiments as Figure 4 on a second putative null allele *daf-9(e1406)*. (A) *daf-9(e1406)* worms start responding to Δ7-DA at 15 hph and require an additional 12–15 h of Δ7-DA for complete adult development. Top, representative colored bars indicating the shift experiment: red bars indicate EtOH carrier and blue bars indicate Δ7-DA. Bottom, histograms indicate proportions of phenotype frequencies between biological replicates ± standard deviations. (B) Pie charts indicate proportions of dauers (red), incomplete adults (yellow), and complete adults (blue) as a function of total amount of time exposed to Δ7-DA (*x*-axis) when exposed to Δ7-DA at different hours post-hatch (*y*-axis). (C) Pulse experiments indicate minimal times necessary for complete development. Top, diagram of pulses used per experiment. Bottom, bar graphs indicate proportions of phenotype frequencies between biological replicates ± standard deviations.
(TIF)

Figure S5 Commitment points of strain AA277 as a function of environmental condition shifts. Top, representative colored bars indicating the experimental paradigm of shift: red bars indicate unfavorable conditions and blue bars indicate favorable conditions. Bottom, bars indicate means of dauer frequencies ± standard deviations between biological replicates. Numbers in parentheses indicate total worms counted per time point.
(TIF)

Figure S6 Ablations of the XXX cells uncouple Δ7-DA production from environmental regulation. (Left) DIC and (right) fluorescent images of AA277 ablated as indicated below. (A) Worms were ablated during L2d and recovered in favorable conditions. (B) L2d ablated worms were let to recover on 10 nM Δ7-DA. (C). Worms were grown to L2d, and XXX cells were ablated after commitment to L3 at 27 hph.
(TIF)

Table S1 Values of q statistic calculated by a Tukey type multiple comparison test for differences among variances. Variances are arranged in ascending order from left to right and from top to bottom. This test takes the difference in natural logarithms of variance values of each time point and normalizes it to a standard error of sqrt(2/(k−1)), where k are the number of biological replicates at each time point. Significant differences in pair-wise comparisons of variances which are larger than q_,0.05,∞,k_ are marked in red and non-significant differences are marked in green. Numbers indicate the computed q statistic. Table S1A shows the computed Tukey type multiple comparison test for differences among variances for shift to growth experiment (Figure 1C), Table S1B shows comparisons for Figure 1C, and Table S1C shows comparisons of *daf-9(dh6)* shift from EtOH to Δ7-Dafachronic acid (Figure 4B).
(RTF)

Table S2 Oligonucleotides used in quantification of *daf-9* transcripts.
(RTF)

Text S1 Supplemental experimental procedures.
(RTF)

## Abbreviations

Δ7-DA: Dafachronic Acid
Cut: Cuticle shedding defect
DA: Dafachronic acid
DAF: Dauer formation defective
E: Ecdysone, 20-E, 20-hydroxyecdysone
hph: hours post hatch
InsR: Insulin Receptor
IPC: Insulin producing cells
JH: juvenile hormone
Mig: Gonad Migratory Defective
PG: Prothoracic gland
SDS: sodium dodecyl sulfate
TGFβ: Transforming Growth Factor–β
