# Supplementary figures and images for: Hormonal Signal Amplification Mediates Environmental Conditions during Development and Controls an Irreversible Commitment to Adulthood
Source: PLoS Biol. 2012 Apr 10;10(4):e1001306. doi: 10.1371/journal.pbio.1001306 (PMC3323525; doi:10.1371/journal.pbio.1001306)

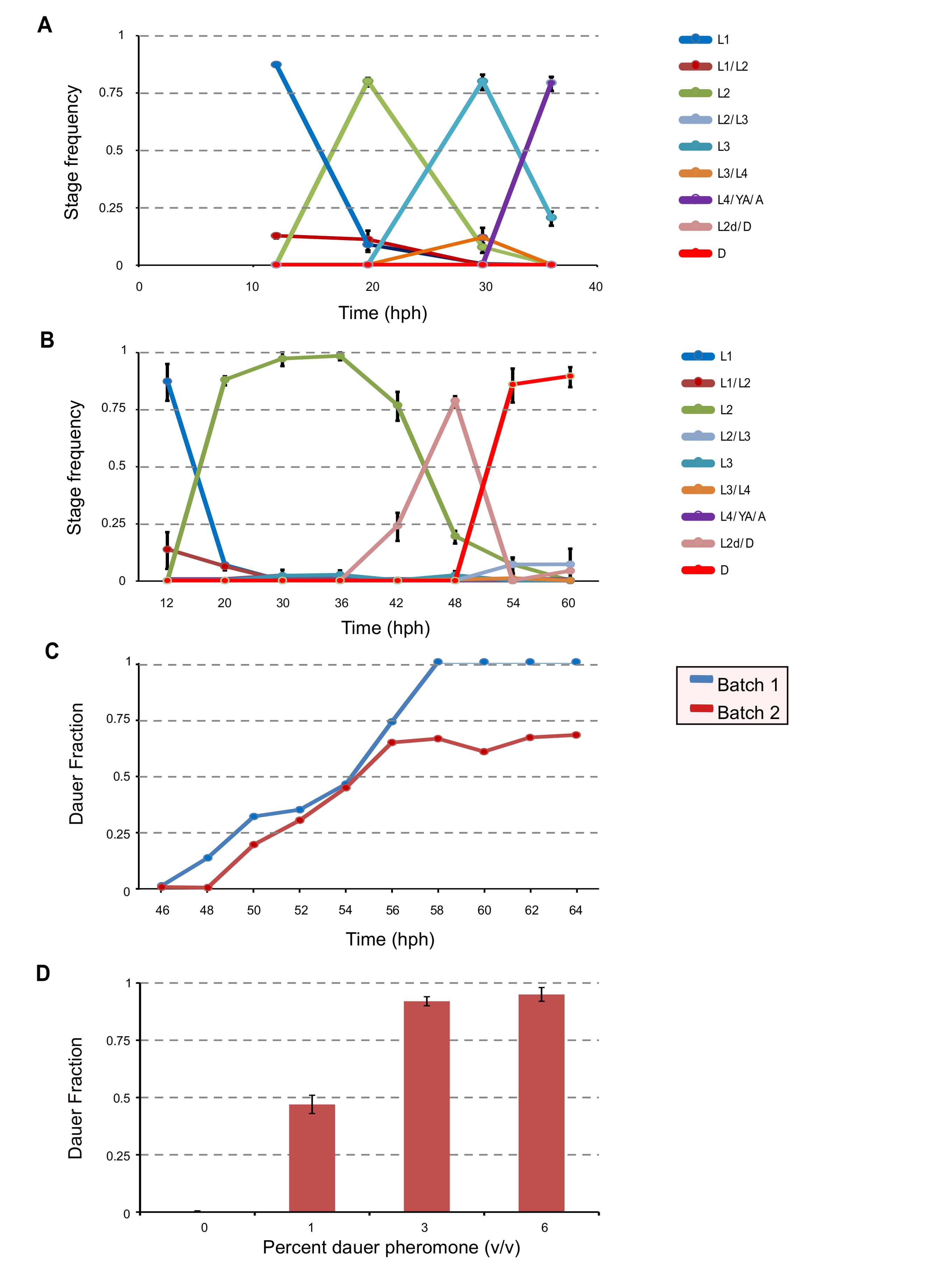

Supplement: Figure S1 — Time course of staged animals grown in environmental conditions. (A) Worms grown in favorable conditions of 7.5 mg/ml HB101 as a bacterial food source, ∼1 worm per µl and 20°C and (B) worms grown in the aforementioned conditions, supplemented with 3% v/v crude pheromone extract. Points indicate means ± standard deviation across three biological replicates. (C) SDS resistance begins 4–6 h after the L2d to dauer molt. Two time courses are indicated using pheromone extracts produced from different worm broods on different days. (D) Percent of dauers formed when pheromone extract was added into growth containing WT worms grown in conditions described above. (TIF) [file pbio.1001306.s001.tif]

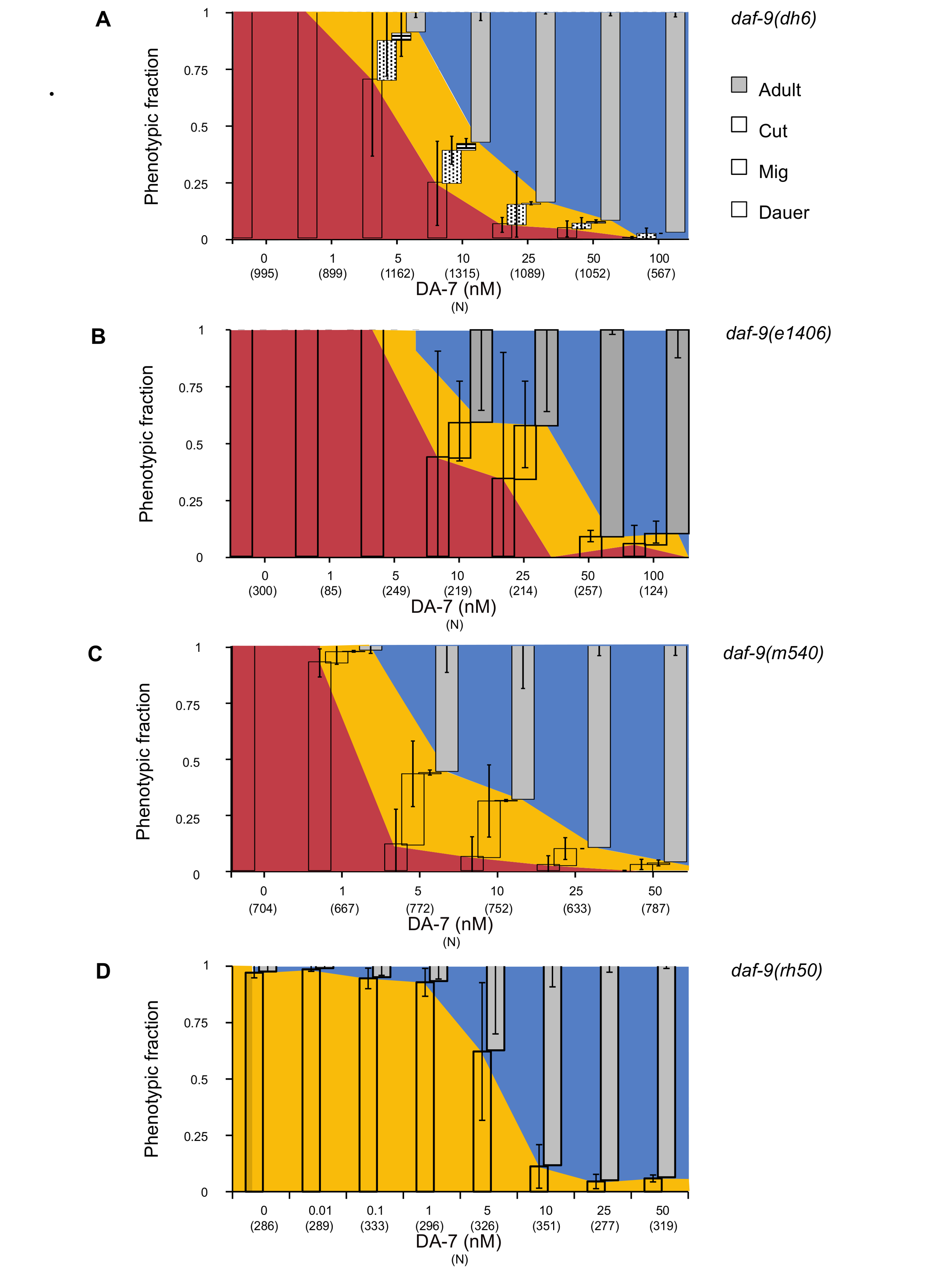

Supplement: Figure S2 — High amounts of DA are required for complete adult development. Distributions of dauer (transparent), Mig (dotted), Cut (hatched), and normal adult (grey) phenotypes when mutants were hatched after a non-synchronous bleach (worms typically hatch over a 15 h window at these growth conditions). (A) daf-9(dh6), (B) daf-9(e1406), (C) daf-9(m540), and (D) daf-9(rh50). Red represents all dauers, yellow represents all abnormal adult phenotypes, and blue represents all complete adults. Bars represent means ± standard deviations across three biological experiments. Numbers in parentheses indicate total worms counted per time point. (TIF) [file pbio.1001306.s002.tif]

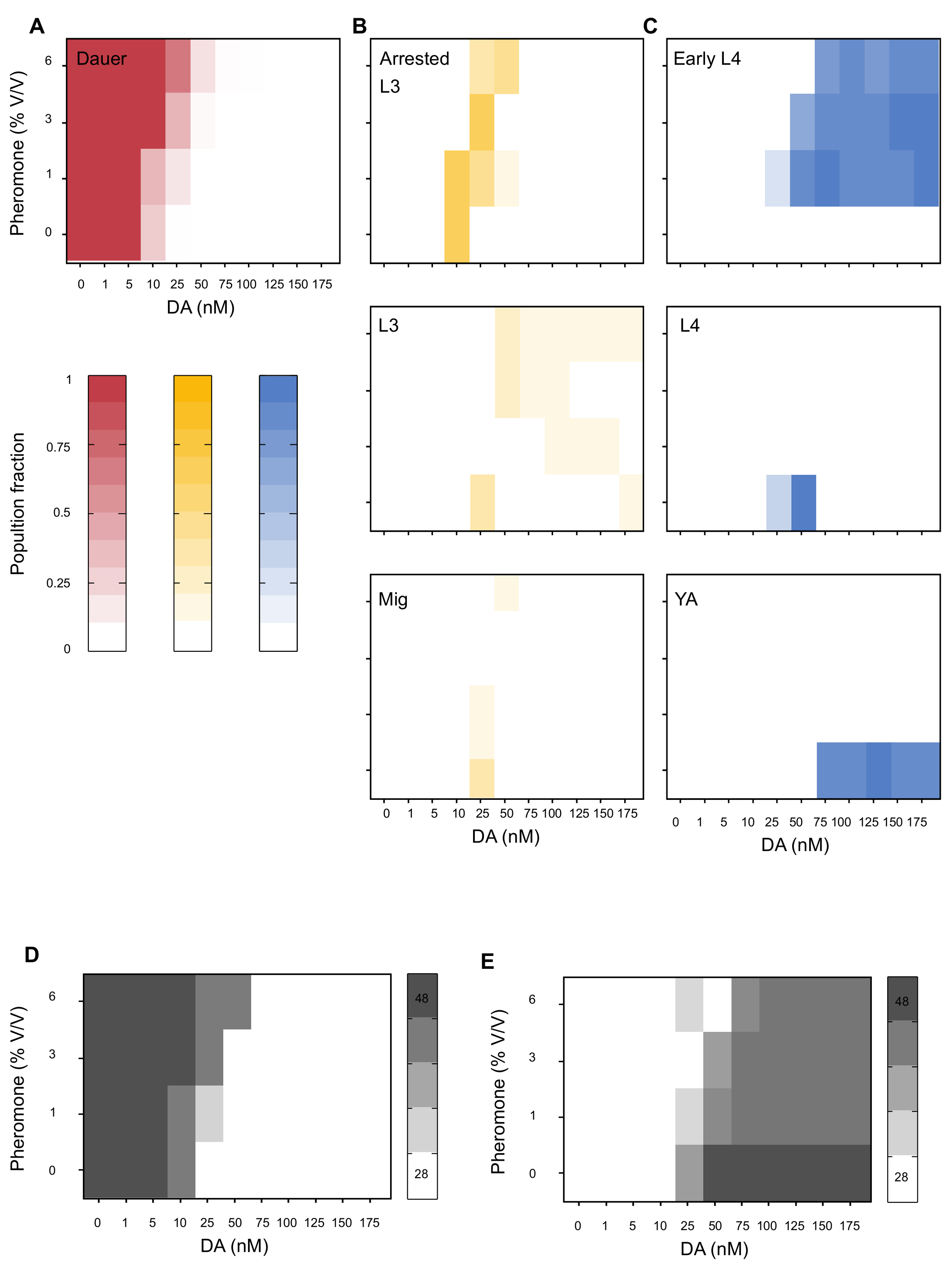

Supplement: Figure S3 — Distribution of developmental stage and phenotype of synchronously hatched daf-9(dh6) in combinations of dauer pheromone and Δ7-DA. (A–C) Color intensity on each pixel on the heat map represents the mean of three biological experiments. For example, the pixel 0% pheromone and 10 nM DA have a population composed of 28.5% dauer, 68.2% arrested L3, and 3.3% Mig. Red, regions dominated by the dauer phenotype. Yellow, areas dominated by the incomplete adult or arrested L3 phenotypes. Blue, areas dominated by the adult phenotypes: early L4, L4, and young adult. Besides the dauer and the arrested L3 stages, all other stages were gravid adults the following day. (D,E) DA controls developmental rate: each pixel is an index composed of a weighted sum of developmental distribution normalized to the maximal growth rate of WT N2 worms. (TIF) [file pbio.1001306.s003.tif]

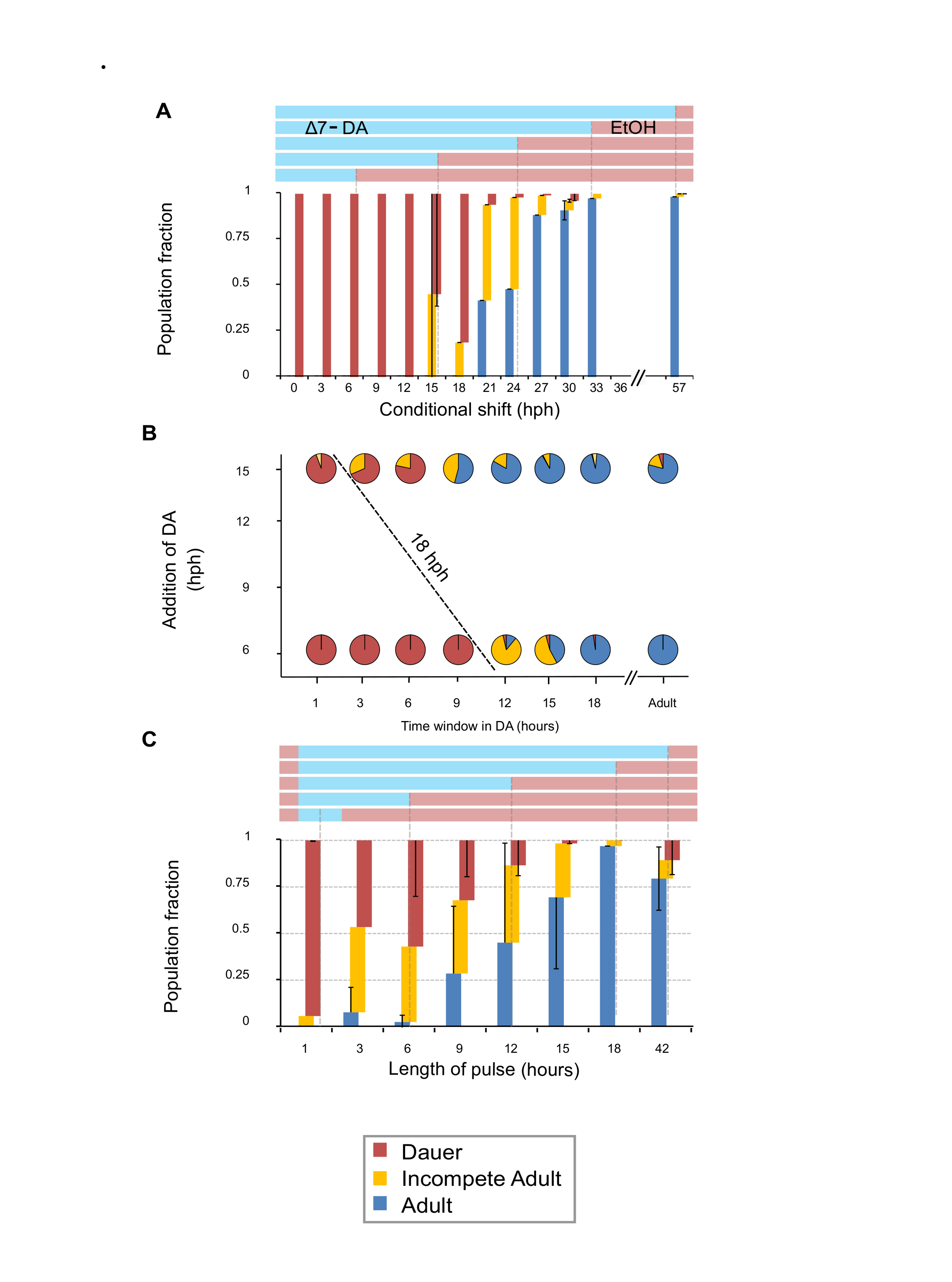

Supplement: Figure S4 — Temporal activity of Δ7-DA. This provides similar experiments as Figure 4 on a second putative null allele daf-9(e1406). (A) daf-9(e1406) worms start responding to Δ7-DA at 15 hph and require an additional 12–15 h of Δ7-DA for complete adult development. Top, representative colored bars indicating the shift experiment: red bars indicate EtOH carrier and blue bars indicate Δ7-DA. Bottom, histograms indicate proportions of phenotype frequencies between biological replicates ± standard deviations. (B) Pie charts indicate proportions of dauers (red), incomplete adults (yellow), and complete adults (blue) as a function of total amount of time exposed to Δ7-DA (x-axis) when exposed to Δ7-DA at different hours post-hatch (y-axis). (C) Pulse experiments indicate minimal times necessary for complete development. Top, diagram of pulses used per experiment. Bottom, bar graphs indicate proportions of phenotype frequencies between biological replicates ± standard deviations. (TIF) [file pbio.1001306.s004.tif]

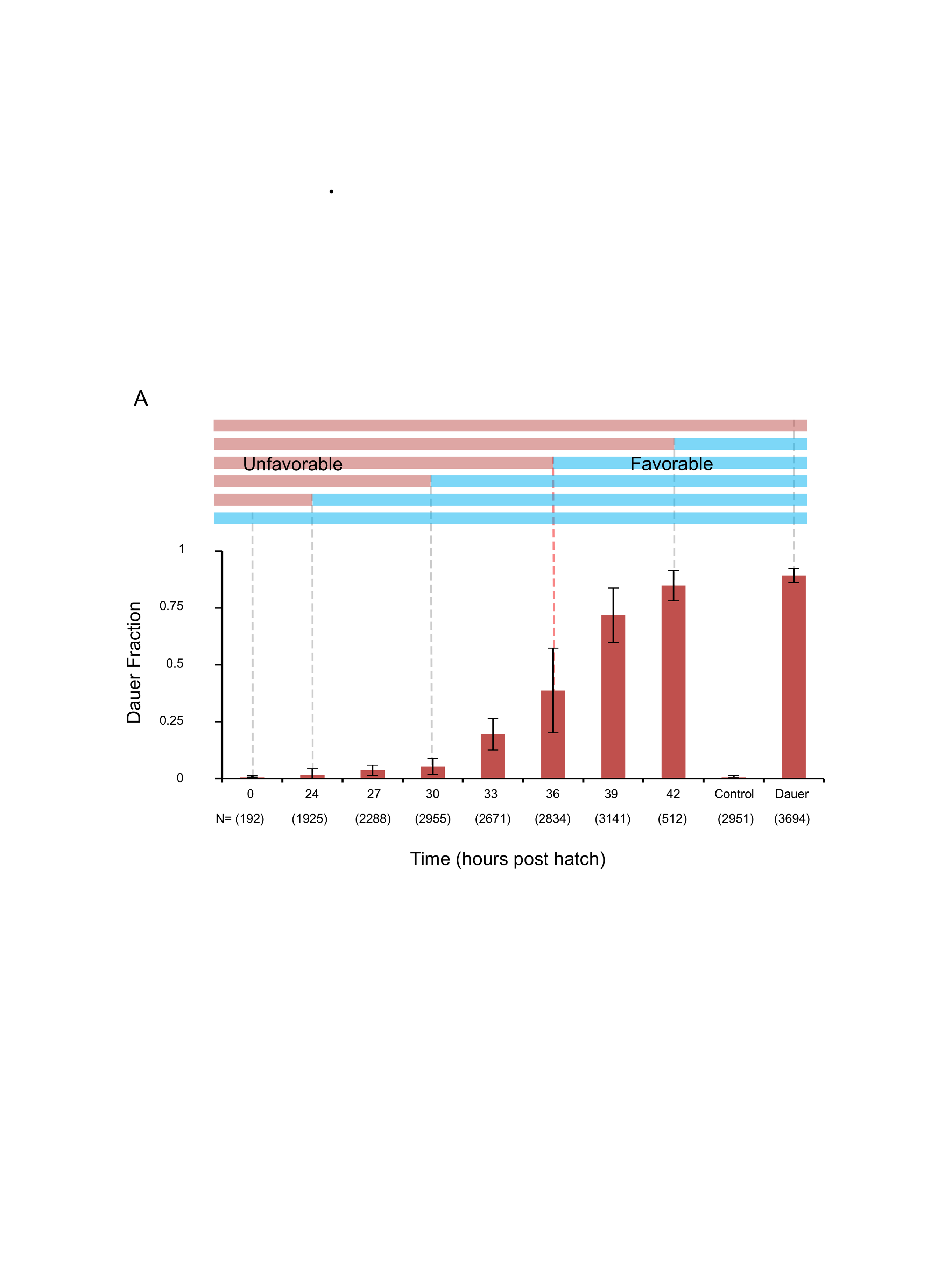

Supplement: Figure S5 — Commitment points of strain AA277 as a function of environmental condition shifts. Top, representative colored bars indicating the experimental paradigm of shift: red bars indicate unfavorable conditions and blue bars indicate favorable conditions. Bottom, bars indicate means of dauer frequencies ± standard deviations between biological replicates. Numbers in parentheses indicate total worms counted per time point. (TIF) [file pbio.1001306.s005.tif]

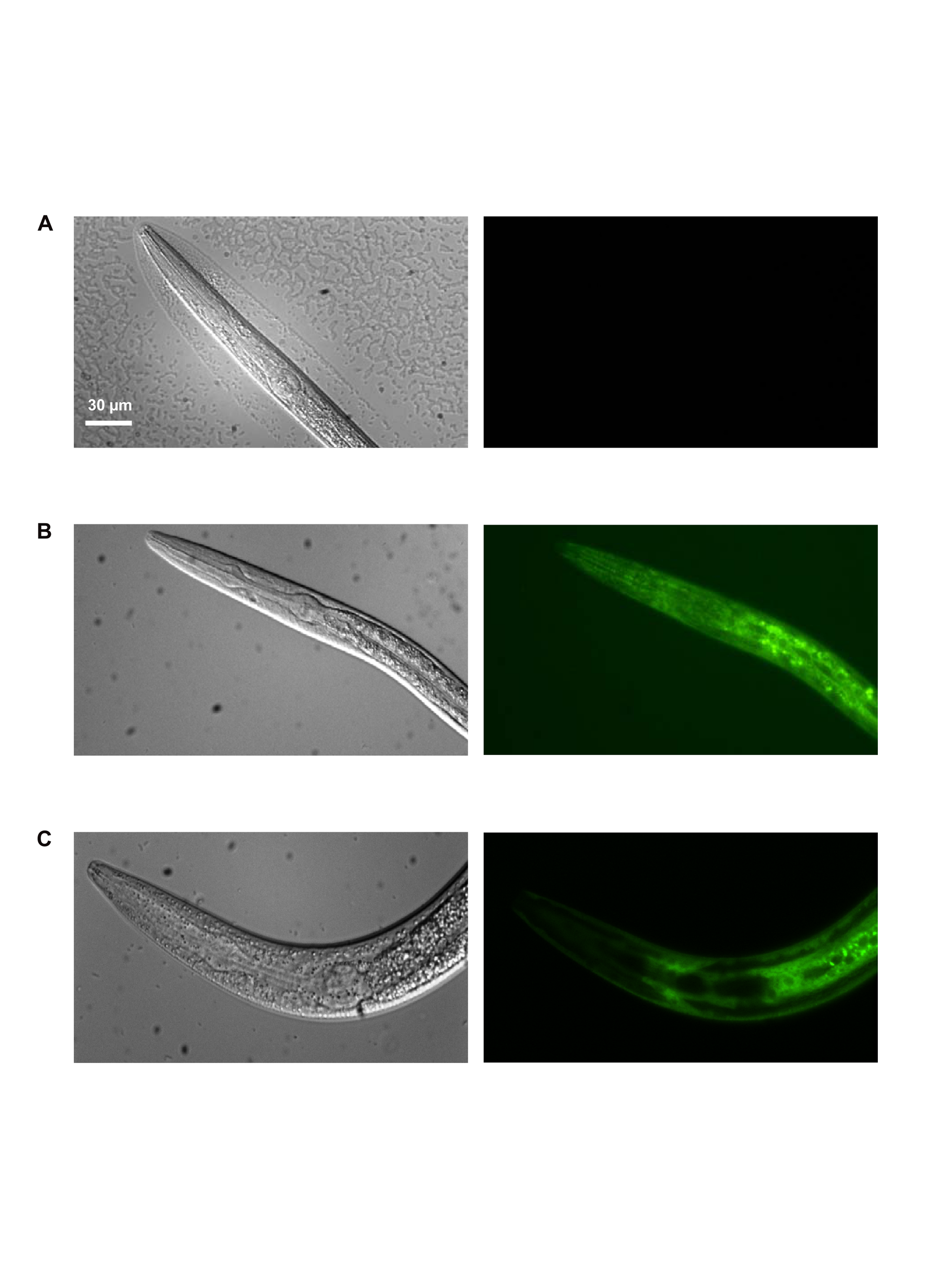

Supplement: Figure S6 — Ablations of the XXX cells uncouple Δ7-DA production from environmental regulation. (Left) DIC and (right) fluorescent images of AA277 ablated as indicated below. (A) Worms were ablated during L2d and recovered in favorable conditions. (B) L2d ablated worms were let to recover on 10 nM Δ7-DA. (C). Worms were grown to L2d, and XXX cells were ablated after commitment to L3 at 27 hph. (TIF) [file pbio.1001306.s006.tif]
