# Supplementary material for: Hormonal Signal Amplification Mediates Environmental Conditions during Development and Controls an Irreversible Commitment to Adulthood
Source: PLoS Biol. 2012 Apr 10;10(4):e1001306. doi: 10.1371/journal.pbio.1001306 (PMC3323525; doi:10.1371/journal.pbio.1001306)
Supplement: Table S1 — Values of q statistic calculated by a Tukey type multiple comparison test for differences among variances. Variances are arranged in ascending order from left to right and from top to bottom. This test takes the difference in natural logarithms of variance values of each time point and normalizes it to a standard error of sqrt(2/(k−1)), where k are the number of biological replicates at each time point. Significant differences in pair-wise comparisons of variances which are larger than q,0.05,∞,k are marked in red and non-significant differences are marked in green. Numbers indicate the computed q statistic. Table S1A shows the computed Tukey type multiple comparison test for differences among variances for shift to growth experiment (Figure 1C), Table S1B shows comparisons for Figure 1C, and Table S1C shows comparisons of daf-9(dh6) shift from EtOH to Δ7-Dafachronic acid (Figure 4B). (RTF) [file pbio.1001306.s007.rtf]

Table S1A	C 	27 	D 	30 	39 	36 	33 	
24 	1.2183 	2.9516 	4.0405 	4.7329 	5.4368 	8.5794	10.1991	
C 		1.7333 	2.8222 	3.5146 	4.2185 	7.3611	8.9808	
27 		` 	1.0888 	1.7812 	2.4851 	5.6277	7.2474	
D 				0.6923 	1.3963 	4.5389 	1.3963 	
30 					0.70392	3.8465	5.4661	
39 						3.1425	4.7622	
36 							1.6196	
B	W24+9 	W24+1 	W24+6 	W24+3 	
W24 	0.4254 	1.2938 	1.3975 	4.0812 	
W24+9 		0.8999 	1.1340 	3.6558 	
W24+1			0.1499 	2.4846 	
W24+6 				2.5217	
C	39 	27 	30 	0 	24 	21 	36 	33 	
None 	-4.1058	-2.83804	-2.33764	-0.5823	0.8135	2.2849	4.2021	5.3723	
39 		1.267766	1.768167	3.5235	4.9193	6.3907	8.3079	9.4781	
27 			0.500401	2.2557	3.6515	5.1229	7.0401	8.2104	
30 				1.7553	3.1511	4.6225	6.5397	7.71	
0 					1.3958	2.8672	4.7844	5.9546	
24 						1.4714	3.3886	4.5588	
21 							1.9172	3.0874	
36 								1.1702	
