# Supplementary material for: Hormonal Signal Amplification Mediates Environmental Conditions during Development and Controls an Irreversible Commitment to Adulthood
Source: PLoS Biol. 2012 Apr 10;10(4):e1001306. doi: 10.1371/journal.pbio.1001306 (PMC3323525; doi:10.1371/journal.pbio.1001306)
Supplement: Table S2 — Oligonucleotides used in quantification of daf-9 transcripts. (RTF) [file pbio.1001306.s008.rtf]

Gene 	Expression 	Spans exons 	Forward Primer 	Reverse Primer 	Amplicon length 	
daf-9 	XXXL/R,    Hypodermis 	9-10 	tcagacgccgtatgtgagag 	gctgggataatctcggtgtt 	140 	
daf-9.1a 	XXXL/R, Hypodermis 	2,4 	aaacgaatgtccagtttggtg 	ttcgaagtcagggaccactt 	183 	
daf-9.1b 	XXXL/R, Hypodermis 	3,4 	tgagagagcctccatttggt 	ttcgaagtcagggaccactt 	157 	
pmp-3 	Intestine 	3,4 	gttcccgtgttcatcactcat   	acaccgtcgagaagctgtaga   	115   	
Y45F10D.4 	Unknown 	1,2 	gtcgcttcaaatcagttcagc   	gttcttgtcaagtgatccgaca   	139 	
ver-2 	ADLL/R 	7,8 	tgtgacattcgccacaaaat 	aaaaactcggcgtttgtttg 	178 	
 
