# Supplementary material for: Hormonal Signal Amplification Mediates Environmental Conditions during Development and Controls an Irreversible Commitment to Adulthood
Source: PLoS Biol. 2012 Apr 10;10(4):e1001306. doi: 10.1371/journal.pbio.1001306 (PMC3323525; doi:10.1371/journal.pbio.1001306)
Supplement: Text S1 — Supplemental experimental procedures. (RTF) [file pbio.1001306.s009.rtf]

Supplemental Experimental Procedures   Strains UsedN2, Bristol, AA111: daf-9(rh50), AA199: daf-9(dh6),daf-9(e1406),AA277: lin-15(n765), dhIs64[daf-9::GFP, lin-15(+)],AA306; lin-15(n765), dhEx94[1,4reg+ex1 daf-9::GFP, lin-15(+)],  DR2281: daf-9(m540)Modifications to synchronized worm hatchAll worms were handled using standard growth and cultivation techniques (Lewis, 1995). Worms were synchronized at the hatch as described by (Baugh et al., 2009) with changes as follows. Embryos of the second bleach were seeded onto 10 cm NGM plates and topped with a 1:1 (v/v) mixture of OP50 and S complete medium (Sulston and Brenner, 1974) and incubated in a 20°C incubator. This drying step helps keeping un-hatched eggs from re-suspending after the wash. Sixteen hours after the bleach, plates were inspected for embryos only and plates containing hatched worms were discarded. Plates were incubated for a further hour and then washed with S basal to suspend all hatched worms. Less than 1% of eggs were found after this wash. Pooled worms were washed once in S complete and used for subsequent experiments. Purification of RNA100 µl of acid washed (1M HCl) sand  was aliquoted into eppendorf tubes. Frozen worms in TRIzol were thawed (Start volume= TRIzol + worms = 1 ml; SV), transferred to sand containing tubes and vortexed vigorously for 10 min. Tubes were allowed to settle at room temperature for 5 min.  20% SV of ChCl3, and TRIzol solution were aliquoted into fresh tubes mixed thoroughly for 15 sec and centrifuged at 12000 RCF for 15 minutes 2-8°c. Aqueous phase was transferred to new tubes mixed with 50% SV of 100% Isopropanol and let precipitate at -20°c overnight. Samples were centrifuged at 12000 RCF for 10 min 4°c, pellet was washed with 1x SV of 75% (v/v) Ethanol (in DEPC-H2O) and EtOH remainders were aspirated. Pellets were resuspended in 10% SV of DEPC treated DDW. Computational worm straightening Each worm was outlined on the DIC image and a central axis was drawn through all bent segments. Pixels on the perpendicular line between the central axis and the worm boundary were recorded and added to the previous section until the entire worm was rendered in a straight alignment. Since each worm had a representative image in DIC and in fluorescence imaging, the same coordinate system per central axis and worm boundary was applied to both images and the same computation was performed to retrieve fluorescence data. Each worm renders an array of coordinate pixels which were transformed into mean grey value (ranging between 0, black to 255, white).     
